# Appearance may be deceiving: Mexican sand flies (Diptera: Psychodidae: Phlebotominae) embrace a high diversity of cryptic species

**DOI:** 10.1093/jisesa/ieaf070

**Published:** 2025-07-25

**Authors:** Yokomi N Lozano-Sardaneta, Herón Huerta, Alejandro Benítez-Guzmán, Jacquelynne B Cervantes-Torres, Atilano Contreras-Ramos

**Affiliations:** Colección Nacional de Insectos, Departamento de Zoología, Instituto de Biología, Universidad Nacional Autónoma de México, Ciudad de México, Mexico; Laboratorio de Entomología, Instituto de Diagnóstico y Referencia Epidemiológicos ‘Dr., Manuel Martínez Báez’, Ciudad de México, Mexico; Departamento de Microbiología e Inmunología, Facultad de Medicina Veterinaria y Zootecnia, Universidad Nacional Autónoma de México, Ciudad de México, Mexico; Departamento de Microbiología e Inmunología, Facultad de Medicina Veterinaria y Zootecnia, Universidad Nacional Autónoma de México, Ciudad de México, Mexico; Colección Nacional de Insectos, Departamento de Zoología, Instituto de Biología, Universidad Nacional Autónoma de México, Ciudad de México, Mexico

**Keywords:** Species delimitation, cytochrome oxidase subunit 1, MALDI-TOF MS, sibling species

## Abstract

Phlebotomine sand flies stand out for their role in vector-borne diseases, having taxonomic priority in aspects of public health. Traditional identification based on morphology involves some limitations that have been corrected with the implementation of complementary methodologies such as cytochrome c oxidase subunit I barcoding and recently mass spectrometry. In Mexico, nearly 38% of sand fly species count with a molecular characterization, but additional information is still necessary for improving sand fly species delimitation. We carried out a molecular species delimitation study of sand flies distributed in the Mexican Transition Zone, between the Nearctic and Neotropical regions, with newly generated cytochrome c oxidase subunit I barcodes, and the first protein profiles created. Compelling evidence showed putative new taxa emerge from *Micropygomyia* aff. *durani* (Vargas & Diaz-Nájera) and *Pintomyia* Series serrana Barretto, and several cryptic species be contained within the genera *Micropygomyia* and *Psathyromyia*, which could be of biological and epidemiological interest. However, for some taxa an exhaustive taxonomic revision at the morphological and molecular levels is recommended, especially for sand flies of wide distribution in the New World.

## Graphical Abstract

**Graphical Abstract:**
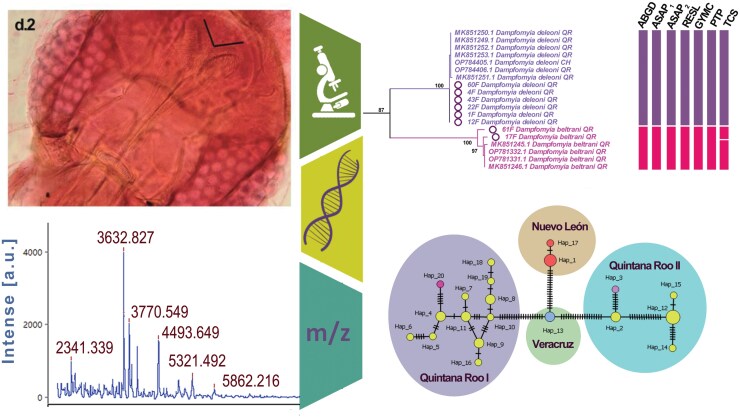

## Introduction

Phlebotomine sand flies (Diptera: Psychodidae: Phlebotominae) are hematophagous insects of great epidemiological relevance for their role as vector-borne diseases, mainly of the protozoan *Leishmania* in at least 99 countries (Akhoundi et al. 2016). Nearly 10% of described sand fly species are incriminated as vectors, bearing high priority in public health. Yet despite their epidemiological role, all phlebotomine species deserve special attention regarding their taxonomic and systematic classification in order to understand their diversity and ecological interactions (Dvorak et al. 2014, Rodrigues et al. 2024a).

Traditional identification of sand flies relies on mounting different parts of the specimens to observe morphological features, which implies having experience and specialized entomological knowledge that result laborious and time-consuming (Contreras-Gutiérrez et al. 2014, Chavy et al. 2019). Furthermore, species-level identification may be hampered by phenotypic plasticity among species or their populations, the absence of differences between closely related taxa, the presence of isomorphic or cryptic species, and incomplete morphological keys for taxa of which only a single-sex has been described (Arfuso et al. 2019, Pinto et al. 2023). Nowadays, barcoding through amplification of the cytochrome c oxidase subunit I (COI) gene, has not only proven a useful method for species-level identification of sand flies, separation of closely related species, and population genetics studies, but has also revealed a large hidden genetic variability of cryptic species, as well as putative new species that need morphological attention in genera such as *Evandromyia*, *Nyssomyia Sergentomyia*, *Phlebotomus*, *Pintomyia*, *Psathyromyia*, and *Psychodopygus*, with several taxa still lacking molecular information (Pinto et al. 2023, Posada-López et al. 2023, Rodrigues et al. 2024b, Soomro et al. 2025).

In the New World, the amount of molecular data has increased exponentially in the last few years, yet new challenges have arisen in establishing phylogenetic relationships due to incongruence between molecular and morphological information, which makes delimitation of sand fly species difficult, especially when there is cryptic diversity (Lozano-Sardaneta et al. 2023a). The implementation of complementary methods could help to solve this problem, for instance, obtaining protein profiles through Matrix-Assisted Laser Desorption/Ionization Time Of Flight Mass Spectrometry (MALDI-TOF MS), proved to be useful for sand fly species identification, blood-meal preferences, and *Leishmania* detection in French Guyana, Ecuador, and Brazil, which could accelerate epidemiological surveillance programs, as it is a fast and inexpensive technique initially proposed for identification of bacteria in clinical diagnostics (Dvorak et al. 2014, Mathis et al. 2015, Arfuso et al. 2019, Chavy et al. 2019, Silva et al. 2024).

Different studies have focused on the Mexican sand fly fauna, with COI-barcodes generated for at least 20 of 58 (38%) species distributed in 5 states, although phlebotomine species have been recorded in at least 24 Mexican states, and therefore considerable molecular information is still necessary to improve species delimitation in the country (Lozano-Sardaneta et al. 2020, 2023a, Rodríguez-Rojas et al. 2024). Since Mexico has a complex biogeographical history, characterized by convergence of the Nearctic (which includes 5 biogeographic provinces) and Neotropical (which includes 9 biogeographic provinces) regions, each with different biotic and abiotic factors that could constraint species distribution within definite areas (Morrone et al. 2002). We aim to carry out formal species delimitation of sand flies distributed in seven Mexican states, located in the Nearctic and Neotropical regions, to define their molecular identity throughout their distribution, and test whether they hide potential cryptic species. For this study, new COI-barcodes were generated, and information previously available for Mexico and other countries for the same species was used to strengthen delimitation analysis, also considering phylogeographic patterns. Additionally, MALDI-TOF MS technique was incorporated as a complementary tool for species identification.

## Materials and Methods

### Collection and Taxonomic Identification of Sand Fly Specimens

The sand fly specimens analyzed were previously collected by Instituto de Diagnóstico y Referencia Epidemiológicos ‘Dr. Manuel Martínez Báez’. Additional monitoring was performed in the localities: (i) Las Adjuntas in Santiago, Nuevo León (04/08/2023; 25° 18′ 03.3480″ N, 100° 08′ 17.1600″ W, 731 m.a.s.l.); (ii) Jardín Botánico Helia Bravo in Zapotitlán, Puebla (11/06/2024; 18° 19.577′ N, 97° 27.048′ W, 1,426 m.a.s.l.); and (iii) Ojo de Agua Grande in Villa Tamazulapam del Progreso, Oaxaca (18/06/2024; 17° 41.358′ N, 97° 33.489′ W, 2,024 m.a.s.l.). Collection was carried out using a Shannon trap during the 6 PM to 11 PM with protected human bait.

For morphological identification, the head, wings, and last segments of the abdomen of female and male sand flies were dissected and mounted using a temporal medium (Lozano-Sardaneta et al. 2023a). The rest of the body was used for the DNA extraction, and MALDI-TOF MS. Taxonomic identification was carried out using actualized keys for sand flies from Mexico (Ibáñez-Bernal 2024).

### DNA Extraction, PCR, and Sequencing

DNA was extracted from abdomen segment using Chelex-100 at 10% (Lozano-Sardaneta et al. 2021). A fragment of ~ 600 bp of the COI was amplified using the primers LCO1490 and HCO2198 (Folmer et al. 1994), following previously standardized PCR conditions (Lozano-Sardaneta et al. 2021). PCR mixture was prepared at 25 μl with: 12.5 μl GoTaq Green Master Mix (Promega, Madison, USA), 1 μl of each primer (100 ng), 2 μl DNA (~60 ng/μl), and 8.5 μl nuclease-free water. Electrophoresis was performed in 1.5% agarose gel stained with 0.2 μl Midori Green Advance DNA stain (Nippon Genetics Europe). Purification and sequencing of PCR products was carried out by Laboratorio de Secuenciación Genómica de la Biodiversidad, Instituto de Biología, UNAM (IB-UNAM).

### Sequences and Phylogenetic Analysis

Sequences obtained were compared at NCBI database using BLASTn (https://blast.ncbi.nlm.nih.gov/Blast.cgi). The accession numbers for the GenBank are from PV367443 through PV367482, and for BOLD systems are from YOK001-25 until YOK040-25 (Supplementary Table S1). The alignment was performed using the ClustalW algorithm in MEGA X (Kumar et al. 2018), including the new sequences generated in this study and additionally GenBank sequences available for COI gene of the same sand fly species, mainly from Mexico, but in case that the information was not available, sequences from other countries were considered.

The substitution model was selected considering the lowest Bayesian Information Criterion that showed a score of 18,797.838 to perform a maximum likelihood (ML) phylogenetic tree, with 10,000 nonparametric bootstraps replicates using General Time Reversible (GTR) + Gamma distribution (+G) + Invariant sites (+I) substitution model in MEGA X. Genetic pairwise distances were calculated with Kimura-2-parameter substitution model in MEGA X.

Also was performed a phylogenetic tree using Bayesian Inference (BI) in BEAST X v10.50 program (Suchard et al. 2018). For designing the analysis and control file the graphical application BEAUti was used (https://beast.community/beauti), with a GTR substitution model (Tavaré 1985) considered the previous ML analysis. The “Coalescent Constant Size” model (Kingman 1982) and “Strict Clock” was selected. The run was performed with 10,000,000 generations (sampling every 1,000). Trace logs were visualized in the Tracer v1.7.2 program, and then a maximum credibility tree (MCC) was generated with retained trees in TreeAnnotator v10.50.0. Finally, FigTreev.1.4.4 program (http://tree.bio.ed.ac.uk/software/figtree) was used to visualize, and edit the tree.

### Species Delimitation Analysis

Species delimitation was performed using the follow algorithms: (i) Automatic Barcode Gap Discovery (ABGD) that it is a fast and simple method to split a sequence alignment into potential species based on barcode gap, which can be run on the web server https://bioinfo.mnhn.fr/abi/public/abgd/abgdweb.html (Puillandre et al. 2012). The input parameters values to construct the matrix were: Pmin = 0.005, Pmax = 0.1, X (relative gap width) = 1.0, with simple distance, and the partition with prior maximal distance of (P) = 0.013 was considered, following the parameters recommended for sand fly species from New World (Pinto et al. 2015, Rodrigues et al. 2024b). (ii) Assemble Species by Automatic Partitioning (ASAP) is a hierarchical clustering algorithm that only uses pairwise genetic distances. It proposes species partitions ranked by a score system (asap-scores, *P*-value and threshold distance) that provides insight into intraspecific diversity (Puillandre et al. 2021). ASAP analysis was running in the web server https://bioinfo.mnhn.fr/abi/public/asap/ using the default parameters and simple distances, the best partition selected was the one with the lowest ASAP-score. (iii) Refined Single Linkage (RESL) employs single linkage clustering as a tool for the preliminary assignment of records to Molecular Operational Taxonomic Units (MOTUs) and a subsequent step that employs Markov Clustering. This algorithm is implemented in the BOLD systems to generate the Barcode Index Numbers. The RESL analysis was performed by constructing a new dataset including our new COI sequences and those processed by other studies in Mexico for the same species, this method was performed directly in BOLD (https://v4.boldsystems.org/index.php/) with the “cluster sequences” tool. (iv) Generalized Mixed Yule-Coalescent (GMYC) it is a likelihood method for delimiting species when only single-locus information is available by fitting intra- and inter-species branching rates on a time-calibrated ultrametric tree (Fujisawa and Barraclough 2013, Zhang et al. 2013). For this method the MCC tree obtained from BEAST was used as input data in the web server https://species.h-its.org/gmyc/, with the “single threshold” method. (v) Poisson Tree Processes (PTP) is simple, fast, and robust model that can delimit species using nonultrametric phylogenies since is a model speciation rate using number of substitutions (Zhang et al. 2013). For PTP analysis was used the ML tree obtained from MEGA as an input file for the web server https://mptp.h-its.org/, using single rate methods, the tree was rooted at the mid-point without out-group included. (vi) TCS haplotype network (https://bioresearch.byu.edu/tcs/) is useful for estimate relationships among organisms that span a wide range of divergence allowing to estimate genealogical relationships among sequences (Clement et al. 2002). The final alignment was used as input file to obtain the genealogy between the specimens using the default parameters.

### Populations Genetics

The number of haplotypes (Hn), polymorphic sites (S), nucleotide diversity per species (π), haplotype diversity (Hd), and average number of nucleotide differences (K), was calculated using DnaSP v5.10 for performing the genetic analysis (Librado and Rozas 2009). Fixation index (*Fst*) was considered to differentiate the genetic structure of the sand fly species recovered using DnaSP v5.10 (Librado and Rozas 2009). The *Fst* is a measure of genetic distance consider the isolation and reduction in breeding, values of 0 represent no genetic differentiation, and 1 complete differentiation, and the intermediate values can be classified as “low”, “moderate”, and “high” genetic differentiation (Duran et al. 2024). For sand flies, the limits of *Fst* values using COI gene have not been formally recognized, but in this study considering the values obtained, the ranges were classified as “low” (*Fst* = 0.00 to 0.29), “moderate” (*Fst* = 0.3 to 0.69), and “high” (*Fst* = 0.7 to 1). PopART (http://popart.otago.ac.nz/) was used to construct a haplotype network graph, through TCS Networks and Minimum Spanning Networks to estimate gene genealogies (Bandelt et al. 1999, Clement et al. 2002).

### MALDI-TOF MS Data Acquisition and Processing

A total of 64 specimens for nine genera and 13 sand fly species were analyzed (Supplementary Table S1) through MALDI-TOF MS using an AUTOF MS 1000 equipment (Autobio, Zhengzhou, China). Most of the sand fly specimens analyzed were collected and preserved in 70% ethanol some time ago, so to obtain a correct extraction of proteins it is recommended to perform a previous rehydration in distilled water during at least 24 h. Therefore, thoraxes with wings and legs were rinsed gradually in distilled water in a 1.5-ml microcentrifuge tube during at least 24 h at room temperature. Later tubes were centrifuged at 13,000 rpm, during 5 min and supernatant was discarded to eliminate the distilled water using a micropipette and left until evaporation. Protein extraction consisted in adding 15 μl of 25% formic acid (Meyer, Mexico) and 15 μl of 50% acetonitrile (Avantor Performance Materials, Baker, Mexico) which was previously prepared in water HPLC (Avantor Performance Materials, Baker, Mexico). After a manual homogenization with a micropipette, the tube was incubated for 10 min. The mix was centrifuged at 13,000 rpm for 2 min, and later 1 μl of the supernatant of each protein extract was deposited onto a steel target plate. Once dried, the deposits were covered with a 1-μl α-cyano-4-hydroxy-cinnamic acid matrix prepared in acetonitrile and trifluoroacetic acid (50:50). A calibrator reagent containing a ribonuclease, myoglobin, and protein extracted from *Escherichia coli* was used for calibration of AUTOF MS.

For the Mass Spectra (MS) analysis with the Autof Acquirer version 2.0.18 software (Autobio, Zhengzhou, China) the default parameters were used. The spectra were acquired in linear mode in the ion-positive mode at a laser frequency of 60 Hz and mass range of 2 to 20 kDa. The data acquired was exported into Autof Analyzer version 1.0.50 software for data spectra analysis.

## Results

### Sand Flies Recovered for Molecular Analysis

For site (i) Las Adjuntas a total of 39 specimens were recovered [20♀ of *Lutzomyia cruciata* (Coquillett), 10♂ and 8♀ of *Psathyromyia shannoni* (Dyar) and 1♀ *Psathyromyia texana* (Dampf)]; from (ii) Jardín Botánico Helia Bravo a total of 13 specimens was collected [11♂ and 2♀ *Micropygomyia durani* (Vargas & Diaz-Nájera)]; and from (iii) Ojo de Agua Grande only one specimen (1♂ *Pintomyia* sp.) was recovered. These localities represent a new record of distribution and new molecular information for these species.

For molecular analysis, a total of 40 COI sequences of 649 bp from 11 sand fly species (8♂ and 32♀) classified in five genera, were retrieved (Supplementary Table S1). The sequence retrieved of *Mi*. *durani*, *Micropygomyia cayennensis maciasi*, and *Pintomyia* sp., are new for science, finally the rest of the sequences each represents a new locality record for sand flies in Mexico. Unfortunately, DNA from specimens of the species *Micropygomyia chiapanensis* (Dampf), *Brumptomyia mesai* Sherlock, *Psychodopygus panamensis* (Shannon), *Trichopygomyia triramula* (Fairchild & Hertig), *Lutzomyia longipalpis* (Lutz & Neiva), and *Psathyromyia carpenteri* (Fairchild & Hertig) did not amplify, and new sequences for the analysis were not possible to obtain, possibly because some specimens are old. Additionally, 101 sequences were downloaded from GenBank, from the same species of interest to complete the species delimitation analysis (Supplementary Table S2). The nucleotide diversity was *π* = 0.13622, no INDELs or stop codons were observed.

Intraspecific variation within the species analyzed ranged between 0% and 3.8%, being the sequences of *Pa. carpenteri* recovered from Mexico (states of Veracruz, Quintana Roo, and Chiapas), Panama and Colombia the ones with the highest value (Supplementary Table S3). Interspecific variability ranged between 3.94% and 23.70%, the species with the lowest value were *Br. mesai* versus (vs.) *Brumptomyia hamata* (Fairchild & Hertig) (3.94%) followed by *Pa. texana* vs. *Pa. carpenteri* (9.52%), meanwhile *Da. beltrani* showed the highest interspecific distance with respect to *Micropygomyia cayennensis cayennensis* (Floch & Abonnenc) (23.70%), *Pa. carpenteri* (23.59%), and *Pintomyia ovallesi* (Ortiz) (23.11%) (Supplementary Table S3).

### Molecular Taxonomy of Sand Fly Species

**Fig. 1.**
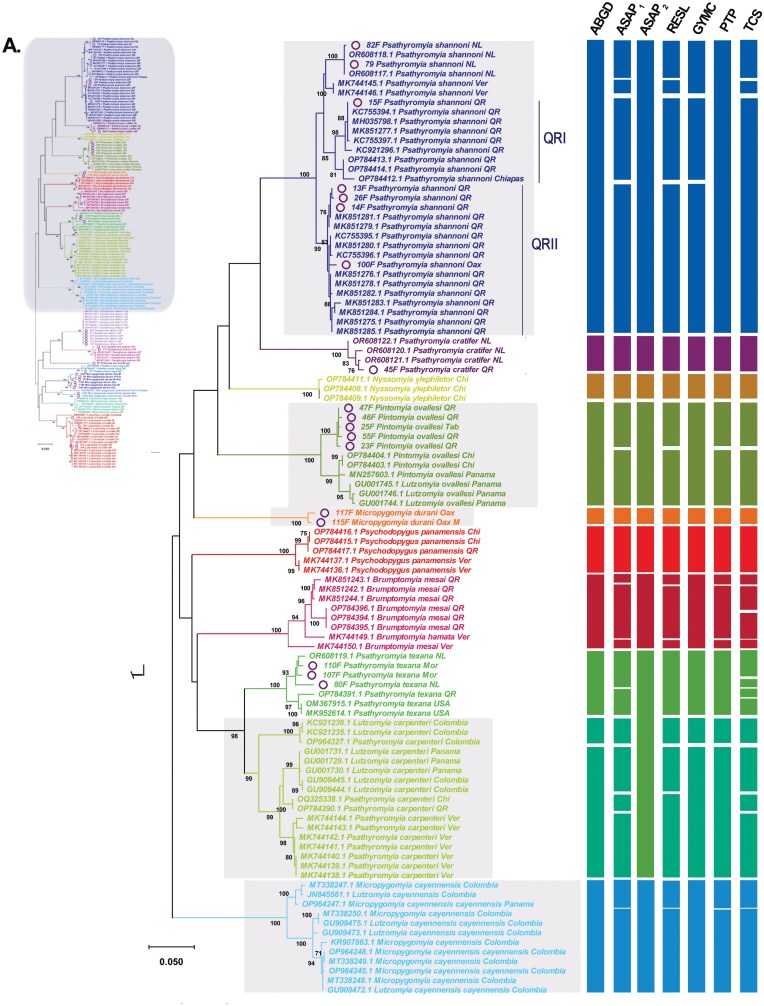
Phylogenetic inference using Maximum Likekihood based on COI sequences from sand flies distributed in Mexico. A) Complete tree; B) phylogenetic tree for 10 sand fly species. On the right side, the delimitation of MOTUs carried out using seven algorithms is differentiated with different colors considering the taxonomic identification. The circle highlights the new sequences from this study. The states from which the specimens were collected are abbreviated as follows: NL = Nuevo León, Ver = Veracruz, QR = Quintana Roo, Chi = Chiapas, Mor = Morelos.

**Fig. 2.**
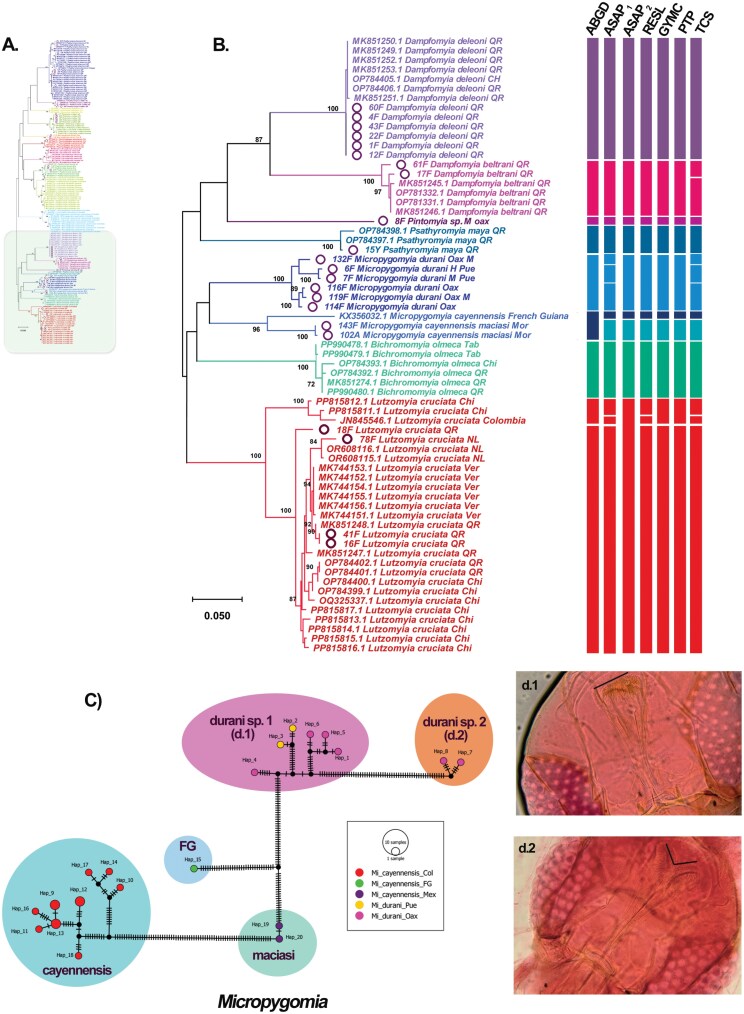
Phylogenetic inference using a Maximum Likelihood based on COI sequences from sand flies distributed in Mexico. A) Complete tree; B) phylogenetic tree for nine sand fly species. On the right side, the delimitation of MOTUs carried out using seven algorithms is differentiated with different colors considering the taxonomic identification. The circle highlights the new sequences from this study. The states from which the specimens were collected are abbreviated as follows: NL = Nuevo León, Ver = Veracruz, QR = Quintana Roo, Chi = Chiapas, Mor = Morelos, Pue = Puebla. C) Haplotype TCS network for species of the genus *Micropygomyia,* lines correspond to mutational steps; dots indicate missing haplotypes and the color circles highlight the species analyzed. Photography d1. and d.2 showed the differences observed between the specimens identified as *Micropygomyia durani*, and the line point out the pharynx differences.

The phylogenetic gene trees generated using BI and ML methods exhibited well-supported clades (posterior probabilities > 0.95; bootstrap > 90) (Figs. 1 and 2; Supplementary Fig. S1). All the sequences obtained in this study clustered with sequences previously recorded in Mexico, confirmed their identity (Figs. 1 and 2). The species *Pa*. *shannoni*, *Pi*. *ovallesi*, and *Pa*. *carpenteri* showed high intraspecific variability (near 3%) that separated the sequences in different clusters (MOTUs), with a bootstrap support of 99% to 100% (Fig. 1; Supplementary Table S3).

The sequences of *Pa*. *shannoni* clustered in 4 different MOTUs, which seems to be related with their geographical distribution: states of Nuevo León (NL, 1 MOTU), Veracruz (Ver, 1 MOTU), and Quintana Roo (QR, at least 2 MOTUs, QRI, and QRII). Considering the ML phylogenetic tree, additional subgroups were considered to compare their interspecific distances, and according to the results *Pa*. *shannoni* NL vs. *Pa*. *shannoni* Ver showed a variability of 2.8%, *Pa*. *shannoni* Ver vs*. Pa*. *shannoni* QR diverge at least 3%, *Pa*. *shannoni* NL vs*. Pa*. *shannoni* QR showed a variability range between 3.8% and 4.6%, and finally the distances between MOTUs distributed within *Pa*. *shannoni* QR ranged between 2.78% to 4.3% (Supplementary Table S4).

A similar comparison was performed for *Pi*. *ovallesi* QR vs. *Pi*. *ovallesi* Panama (Pan) that showed a difference of 5.21%, while in Mexico *Pi*. *ovallesi* QR vs. Chiapas (Chi) recorded a interspecific distance of 4.23% (Fig. 1; Supplementary Table S4). The male specimen from Ojo de Agua Grande, Oaxaca showed similar characteristics with other species of Series serrana, which could be confused with *Pintomyia serrana* (Damasceno & Arouck) (Pereira et al. 2019, Ibáñez-Bernal 2024). However, the sequences of *Pi*. *serrana* from Brazil did not clustered with our sequences, displaying a genetic distance ranging between 17.93% and 20.65%, which suggests that this specimen may represent a putative new species (Supplementary Table S4; Supplementary Fig. S2). Although, a formal description would still be needed later, it may highlighted that the specimen showed some unique traits, for instance a gonostylus with 3 stout spines (1 apical and 2 dorsolateral inserted at the same level), with 1 spine very thin on the basal half of the gonostylus and a subterminal seta in front of the apical spines, gonocoxtie with a basal tuft of 10 diagonal curved rows, paramere simple and widened at distal third, wing with vein R1 ending about level with origin of R2, flagellomeres with simple ascoids.

Although no new sequences of *Pa*. *carpenteri* were obtained, available information for this species was analyzed, displaying a split in 2 MOTUs with an interspecific distance of 7.4%. (Fig. 1) One MOTU is only distributed in the locality Caldas, Colombia (KC921236, KC921235, OP964327), and the second MOTU are distributed in Colombia (Sucre: GU909444, GU909445), Panama (Barro Colorado: GU001729, GU001730, GU001731), and Mexico. The first MOTU mentioned displays an interspecific distance between 6.8% and 7.14% regarding sequences of *Pa*. *carpenteri* from Mexico [QR (OP784390), Chi (OQ325338), and Ver (MK744138 - MK744144)], but the interspecific distance between the sequences from Mexico and Colombia (Sucre) of the second MOTU was 3% (Supplementary Table S3). About the sand fly *Pa. texana*, despite comparing few sequences, their genetic variation ranged between 1.13% and 5.38%, with highest variability observed in sequences from NL compared to sequences from USA (3.94%) and QR (5.38%) (Fig. 1; Supplementary Table S4).

The genus *Micropygomyia* also showed high distance values between the analyzed species. The highest difference was observed between *Mi*. *cay. cayennensis* distributed in Colombia and Panama with respect to *Mi*. *cayennensis* from French Guyana (FG), with a distance of 18.38%, this was followed by *Mi*. *cay. maciasi* from Mexico with a 16.55% distance with reference to *Mi*. *cay. cayennensis* (Col/Pan) (Supplementary Table S3 and S4). Finally, the distance between *Mi*. *cay. maciasi* vs. *Mi*. *cayennensis* FG was 10.20%, clustering in a same clade with a bootstrap value of 96%, unlike *Mi*. *cay. cayennensis* (Col/Pan) which is placed entirely as a separate clade (Fig. 2). The clade of *Mi*. *cay. cayennensis* (Col/Pan) separates into 2 MOTUs (Col I/Pan and Col II) with an interspecific distance of 4.5% between them, which may suggest they represent cryptic species (Fig. 2; Supplementary Table S4; Supplementary Fig. S1). Considering the high genetic distance, results suggest that *Mi*. *cay. cayennensis* (Col I/Pan and Col II), *Mi*. *cay. maciasi* (Mex), and *Mi*. *cayennensis* FG may each represent distinct species, which supports that subspecies should be raised to species status, but corresponding hypotheses require a deep morphological revision.

For the species *Mi*. *durani*, surprisingly the sequences obtained split into two putative species in both phylogenetic analysis (BI and ML) with an interspecific distance of 13.16% (Fig. 2; Supplementary Table S3; Supplementary Fig. S1). The specimens analyzed of sp1 and sp2 (including females and males) showed similar morphology and were collected in Las Cuevas, Santo Domingo de Morelos, Oaxaca, 3 December 2019, but in Cerro Gordo, Santa Maria Tonameca, Oaxaca, 2 December 2019, only was recovered the sp1. Males showed a gonostyle with 5 spiniform setae arranged 2 + 1 + 2; gonocoxite without basal tuft, aedeagal ducts with widened apices and lanceolate shape. Females, showed a pharynx with conspicuous spines on distal portion, the cibarium with at least 20 posterior teeth, and spermatheca pyriform with rings and striations at its junction with individual spermathecal duct. Both sexes showed a wing with vein R1 ending about level with origin of R2, flagellomeres with simple ascoids (Ibáñez-Bernal 2024). The main differences in one of the putative new species showed a subtle difference in the shape of the pharynx. Considering the original description of the nominal species *Mi*. *durani* (in this study labeled as sp. 1), its pharynx has conspicuous distal spines following its rounded structure along a single line, while in the species we refer to as *Mi*. aff. *durani* (sp. 2), its pharynx is wider with conspicuous spines on distal portion, forking in the middle giving a heart-like appearance (Fig. 2 d.2). A formal morphological description of *Mi*. aff. *durani* sp. 2 is not currently possible, because only 2 specimens (1♀ and 1♂) were fortuitously recovered and their morphological similarity with *Mi*. *durani* sp. 1 challenges a detailed description, with additional specimens being necessary.

According to the ML tree the sequences of *Mi*. *durani* sp. 1 and *Mi*. aff. *durani* sp. 2 did not cluster together (Fig. 2), although they agree with the morphological characteristics of the genus *Micropygomyia*. However, *Mi*. *durani* sp. 1 (from Oaxaca and Puebla) clustered with other *Micropygomyia* species (Fig. 2). On the contrary, in the BI tree both putative species (sp.1 and sp. 2) clustered together, yet the other *Micropygomyia* species clustered in different clades (Supplementary Fig. S1). Although, at morphological level *Mi*. *durani* sp. 1 and *Mi*. aff. *durani* sp. 2 could be cryptic species, according to their high interspecific distance and the separation observed in ML and BI analyses it should be assumed these sequences represent species status entities. This molecular evidence, also suggests that the genus *Micropygomyia* Series cayennensis may hide a species complex, and it would be highly advisable to test this hypothesis through complementary morphological and molecular analyses.

The rest of the analyzed species did not show higher genetic differences with respect to previously recorded information in GenBank, except for *Lu. cruciata* from Mexico (NL, Chi, QR, and Ver) that showed high genetic variability regarding a sequence from Colombia (JN845546), and with 2 sequences from Chiapas, Mexico (PP815811 and PP815812) with a variation that ranged between 7.4% and 9.3%, while the variability between sequences from Mexico ranged from 0.16% to 3%, being the specimens of *Lu. cruciata* from Chiapas (0.32% to 2.5%) and Quintana Roo (1.64% to 3%) which showed the highest variability (Supplementary Table S3).

Species delimitation using a fragment of the COI gene partially agrees with the phylogenetic inferences (BI and ML) analyzed in this study. The models ABGD and ASAP_2_ were the most conservative and identified 20 and 18 MOTUs, respectively, however in the case of ASAP_2_ it considered the nominal species *Pa*. *texana* and *Pa*. *carpenteri* as a single nominal species despite their interspecific distance is 9.5%, and something similar occurs with *Mi*. *cayennensis* FG and *Mi*. *cay*. *maciasi* (10.5%) (Figs. 1 and 2). In this study, the lowest ASAP-score was 4.0, but 2 partitions with the same score was obtain, one with distance threshold of 0.023237 that identified 34 MOTUs, and other with distance threshold of 0.080128 that identified 18 MOTUs. On the contrary, algorithms RESL and TCS divide the dataset in 30 and 37 MOTUs, respectively, suggesting high intraspecific variability inside of the species analyzed, which does not agree with the evidence of the phylogenetic analysis or genetic distances; therefore, for this analysis might not be useful for species delimitation. However, the models GYMC (23 MOTUs) and PTP (25 MOTUs) seem complementary to each other and more suitable for species delimitation, therefore were the models considered for population genetics analysis. In the case of the PTP model, it highlights that the genus *Brumptomyia* could include potential cryptic species, in this analysis the only 2 species distributed in Mexico (*Br. mesai* and *Br. hamata*) were included, with the populations of *Br. mesai* from QR vs Ver showing an interspecific distance of 4.6% (Supplementary Table S4), this MOTUs separation also was proposed with the model ASAP_1_, RESL, and TCS, although in the GYMC, ASAP_2_ and ABGD models *Br. mesai* and *Br. hamata* are considered the same species (Fig. 1). Further investigation into the classification of the genus *Brumptomyia* in Mexico is also necessary.

### Population Genetics of Sand Flies

**Fig. 3.**
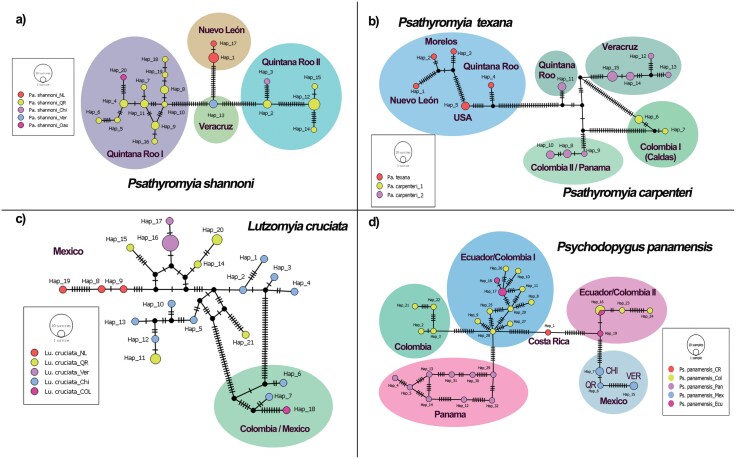
Haplotype TCS network for Phlebotominae species from Mexico. Lines correspond to mutational steps; dots indicate missing haplotypes and the color circles highlight potential cryptic species. A) *Psathyromyia shannoni*, B) *Psathyromyia texana* and *Psathyromyia carpenteri*, C) *Lutzomyia cruciata*, and D) *Psychodopygus panamensis*. The States of specimens collection are abbreviated as follows: NL = Nuevo León, Ver = Veracruz, QR = Quintana Roo, Chi = Chiapas, Mor = Morelos. Countries of GenBank sequences are abbreviated as follows: Col = Colombia, CR = Costa Rica, Ecu = Ecuador, Mex = Mexico, Pan = Panama, USA = United States of America.

**Fig. 4.**
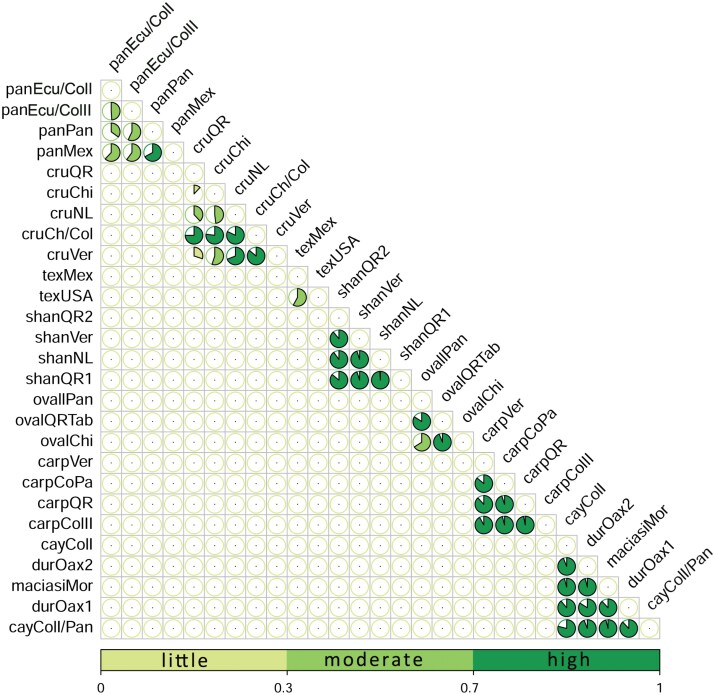
Correlation plot represented as pie chart for fixation index *Fst* values to differentiate the genetic structure of the sand fly species. *Fst* values ranging from 0 (no genetic differentiation) to 1 (complete differentiation). Three ranges were classified as “low” (*Fst = *0.00 to 0.29), “moderate” (*Fst = *0.3 to 0.69), and “high” (*Fst = *0.7 to 1). The species are abbreviated as follows: cru = *Lutzomyia cruciata*, cay = *Micropygomyia cayennensis cayennensis*, dur = *Micropygomyia durani* (sp. 1 and sp. 2), maciasi = *Micropygomyia cayennensis maciasi*, oval = *Pintomyia ovallesi*, shan = *Psathyromyia shannoni*, tex = *Psathyromyia texana*, car = *Psathyromyia carpenteri*, pan = *Psychodopygus panamensis*. The locations are abbreviated as follows: NL = Nuevo León, Ver = Veracruz, QR = Quintana Roo, Chi = Chiapas, Mor = Morelos, Col = Colombia, Ecu = Ecuador, Mex = Mexico, Pan = Panama, USA = United States of America. For more details see Fig. 3 and Supplementary Table S5.

A total of 105 haplotypes (Hd = 0.99303; π = 0.13563; S: 254) were obtained for the sequences analyzed. *Psathyromyia shannoni* was the species with the high number of haplotypes (Hn = 31, Hd = 0.96774), followed by *Lu. cruciata* (Hn = 21, Hd = 0.96581). The rest of the species ranged between 2 and 6 haplotypes with Hd ranged between 0.33333 and 1.0 (Table 1). The species that have unique sequences was not considered in this analysis. The sand flies *Pa. shannoni*, *Pa. texana*, *Pa. carpenteri*, *Lu. cruciata*, *Pi. ovallesi*, and species of *Micropygomyia*, were the species that could include potential cryptic species according to the results of TCS networks (Figs. 2C and 3), and values of fixation index *Fst* (Fig. 4; Supplementary Table S5).

**Table 1.** Genetics differentiation between populations of phlebotomine species distributed in Mexico.

Species	*n*	S	Hn	Hd	K	π	π JC
(1) *Bichromomyia olmeca* (QR-Tab-Ch)	6	12	5	0.93333	5.2	0.00833	0.0084
(2) *Brumptomyia mesai* (QR)	6	15	4	0.8	8	0.01282	0.01298
(3) *Dampfomyia beltrani* (QR)	6	15	4	0.8	5.46667	0.00876	0.00885
(4) *Dampfomyia deleoni* (QR-Ch)	13	2	3	0.61538	0.69231	0.00111	0.00111
(5) *Lutzomyia cruciata* (NL)	3	9	3	1	6	0.00962	0.0097
(6) *Lutzomyia cruciata* (Ch)	8	21	8	1	8.10714	0.01299	0.01313
(7) *Lutzomyia cruciata* (QR)	7	26	5	0.90476	10.85714	0.01740	0.01767
(8) *Lutzomyia cruciata* (Ver)	6	1	2	0.33333	0.33333	0.00053	0.00053
(9) *Lutzomyia cruciata* (Col-Chi)	3	17	3	1	11.66667	0.01870	0.01894
(10) *Micropygomyia durani* sp.1 (Oax-Pue)	6	33	6	1	16	0.02564	0.02619
(11) *Micropygomyia* aff. *durani* sp. 2(Oax)	2	6	2	1	6	0.00962	0.00968
(12) *Micropygomyia cay. cayennensis* (Col-Pan)	3	8	3	1	5.33333	0.00855	0.0086
(13) *Micropygomyia cay*. *cayennensis* (Col)	9	17	6	0.91667	6	0.00962	0.0097
(14) *Micropygomyia cay. maciasi* (Mor)	2	1	2	1	1	0.0016	0.0016
(15) *Nyssomyia ylephiletor* (Chi)	3	2	2	0.66667	1.33333	0.00214	0.00214
(16) *Pintomyia ovallesi* (Pan)	4	13	4	1	6.66667	0.01068	0.01079
(17) *Pintomyia ovallesi* (QR-Tab)	5	7	5	1	3	0.00481	0.00483
(18) *Psathyromyia cratifer* (NL-QR)	4	14	4	1	7.66667	0.01229	0.0124
(19) *Psathyromyia carpenteri* (Col)	3	3	2	0.66667	2	0.00321	0.00322
(20) *Psathyromyia carpenteri* (Ver)	7	12	4	0.80952	4.28571	0.00687	0.00692
(21) *Psathyromyia carpenteri* (Col-Pan)	5	3	3	0.8	1.6	0.00256	0.00257
(22) *Psathyromyia carpenteri* (Chi-QR)	2	0	1	0	0	0	0
(23) *Psathyromyia maya* (QR)	3	9	3	1	6	0.00962	0.0097
(24) *Psathyromyia shannoni* (NL)	4	1	2	0.5	0.5	0.0008	0.0008
(25) *Psathyromyia shannoni* (QR-Ch)	3	7	2	0.66667	4.66667	0.00748	0.00754
(26) *Psathyromyia shannoni* (QR)	6	7	3	0.6	2.33333	0.00374	0.00376
(27) *Psathyromyia shannoni* (QR-Oax)	16	19	12	0.96667	4.63333	0.00743	0.00748
(28) *Psathyromyia texana* (NL-Mor)	4	20	4	1	10.66667	0.01709	0.01731
(29) *Psathyromyia texana* (QR-USA)	3	11	2	0.66667	7.33333	0.01175	0.01189
(30) *Psychodopygus panamensis* (QR-Ch-Ver)	5	11	3	0.8	6.6	0.01058	0.0107
total	153	254	105	0.99303	84.51729	0.13544	

*n*, number of sequences; S, number of polymorphic/indel/missing sites; Hn, number of haplotypes; Hd, haplotype diversity; K, average number of nucleotide differences; π, nucleotide diversity.

The values of *Fst* for species of the genus *Micropygomyia* were rather high (*Fst *= from 0.78 to 0.96), indicating broad genetic structure of the populations of *Mi*. *cay*. *cayennesis* distributed between Colombia and Panama, *Mi*. *cay*. *maciasi* from Mexico, and confirming the genetic differentiation between *Mi*. *durani* (sp. 1) and *Mi*. aff. *durani* (sp. 2) (Figs. 2C and 4; Supplementary Table S5). For the genus *Psathyromyia*, the populations of *Pa. shannoni* distributed in Mexico showed a high genetic structure at the state level (NL, Ver, QR), but highlights that in the state of Quintana Roo 2 populations (QRI vs QRII with a *Fst* of 0.86) are differentiated, which confirms the presence of cryptic species or distinct genetic lineages for this species in Mexico (Figs. 3A and 4; Supplementary Table S5). Something similar occurs in *Pa. carpenteri*, which also showed high genetic structure across its distribution (Figs. 3B and 4; Supplementary Table S5). Although *Pa. texana* was split into 2 clusters, its genetic structure was moderate with *Fst* = 0.58. The genetic structure of the *Lu. cruciata* populations distributed in Mexico (NL, Ver, QR, and ChiI) was low or moderate, with *Fst* values ranging from 0.12 and 0.69, but when comparing these populations with the sequence from Colombia and 2 sequences from Chiapas (ChiII) a high genetic structure was observed, with *Fst* values ranging between 0.77 and 0.86 confirming the presence of cryptic species (Figs. 3C and 4; Supplementary Table S5).

On the other hand, the populations of *Pi*. *ovallesi* have a high genetic structure, and at least in Mexico, there seems to be 2 populations: one distributed across Quintana Roo and Tabasco, and other distributed in Chiapas. It is also important to highpoint that the Mexican populations are completely different from those distributed in Panama (Fig. 4; Supplementary Table S5). Finally, the population structure of *Ps*. *panamensis* was moderate, with *Fst* values ranging from 0.35 to 0.67. Sequences from Mexico had the highest *Fst* values compared to the sequences from Colombia, Ecuador and Panama. Although molecular information about this species is scant, the results obtained may suggest potential cryptic species (Figs. 3D and 4; Supplementary Table S5). Since this species is an important vector of *Leishmania* in the New World, complementary information is needed for better species delimitation.

### MALDI-TOF MS of Sand flies

**Fig. 5.**
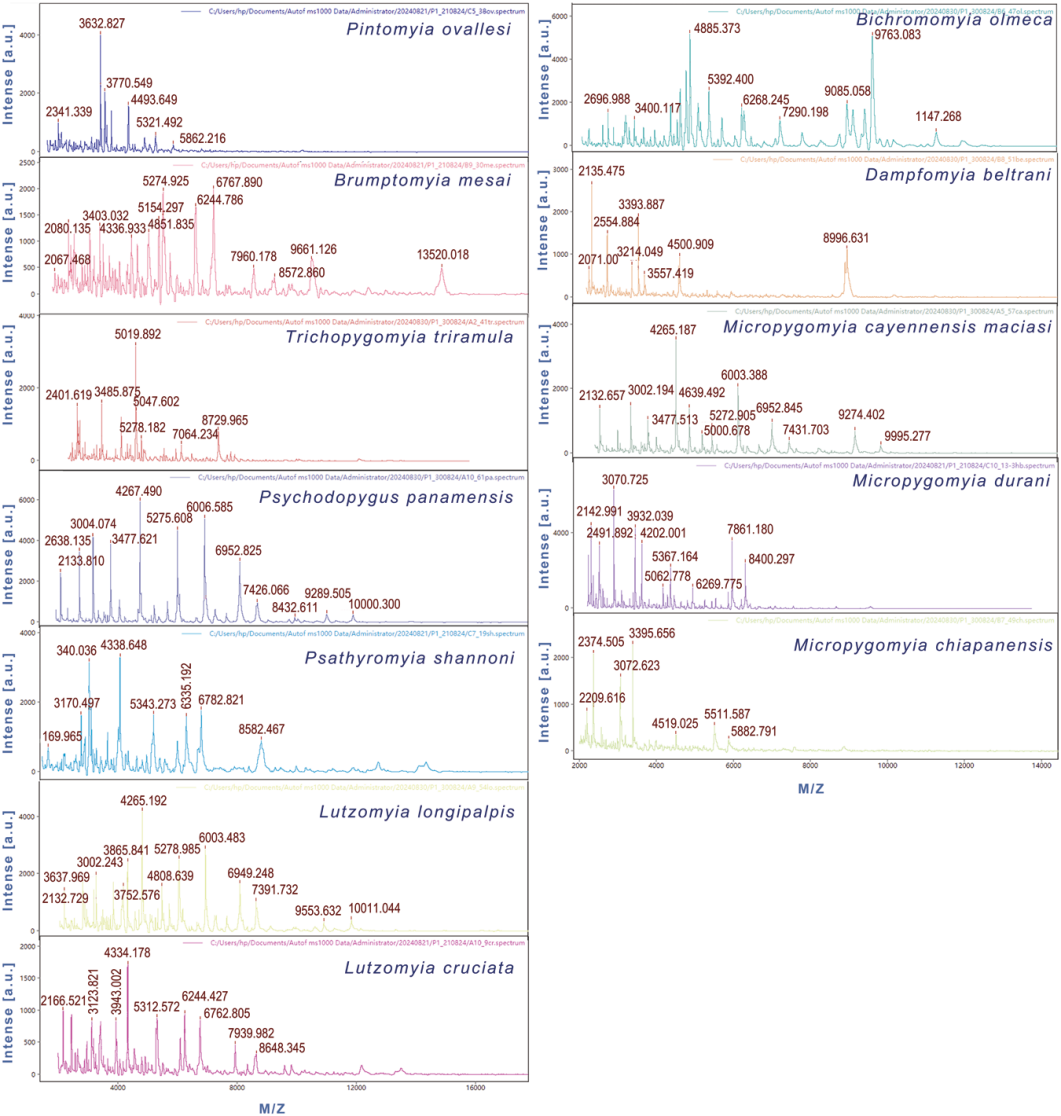
Mass spectrum protein profile from thorax of different Mexican sand fly species. Annotations of spectra represent mass peaks in Daltons. The axis of the Xs showed the *m*/*z*, mass to charge ratio.

**Fig 6.**
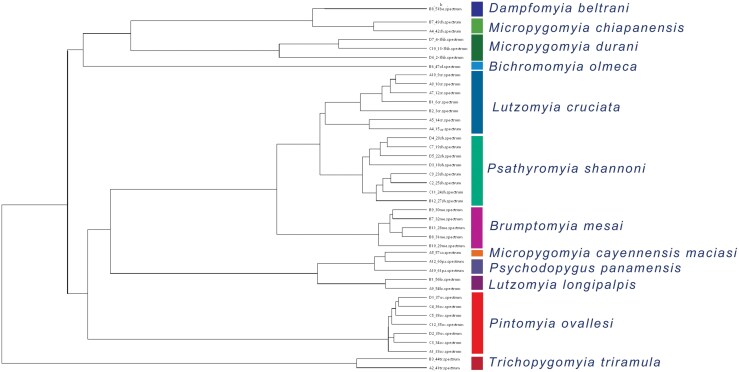
Dendrogram obtained from MALDI-TOF MS constructed from the profile of 12 Mexican sand fly species.

MALDI-TOF MS analysis was performed for 13 species, but only 42/64 available specimens showed reliable and repeatable identification. The spectra showed good resolution and intensity, with wide mass/charge intervals that range from 2 to 6 kDa, representing the first protein profiles for the analyzed species (Fig. 5). For the species *Lu*. *cruciata*, *Pi*. *Ovallesi*, and *Pa*. *shannoni* the largest number of specimens were analyzed, and most of the spectra were homogeneous showing a similar profile that clustering together in the dendrogram (Fig. 6). The spectra of *Ps*. *panamensis* and *Mi. cay. maciasi* cluster together in the dendrogram probably due to their similarities, but the first listed species showed a longer mass interval regarding the other species. Three species for the genus *Micropygomyia* were analyzed (*Mi*. *chiapanensis*, *Mi*. *cay. maciasi*, and *Mi*. *durani* sp1.) but no species cluster together in the dendrogram despite belonging to the same genus. For *Pa*. *shannoni* and *Mi*. *durani* sp1., males and females specimens were included, which did not present differences, both sexes clustering together in the dendrogram, while for the other species only females were analyzed (Supplementary Table S1). This is a preliminary application of MALDI-TOF MS, with results that support this may be a helpful technique for species identification, however not necessarily reflecting phylogenetic relatedness. The biggest challenge we faced for the implementation of this method was obtaining a successful protein extraction, which was probably a consequence that the specimens were preserved in ethanol for several years before processing. For this reason, we were unable to perform COI barcode amplification and MALDI-TOF simultaneously on the same specimens. Complementary protein profiles are still needed to strengthen these first results, including specimens collected from different geographic areas, considering previously reported genetic variability to confirm that the technique may reliably distinguish cryptic species.

## Discussion

In the last 10 years, through an integrative taxonomy approach, it has been possible to elucidate taxonomic questions and establish a correct species delimitation, discrimination between close species, and discover hidden diversity in cryptic species (Contreras-Gutiérrez et al. 2014, Pinto et al. 2023, Rodrigues et al. 2024b). Particularly, DNA barcode analysis has been useful when both sexes are included to separate species, since it is easier to perform a correlation between males with females that are indistinguishable due to their morphological similarities, for example females of the genus *Brumptomyia* or *Trichopygomyia* (Pinto et al. 2015, Lozano-Sardaneta et al. 2020, Soomro et al. 2025). This study performs species delimitation of Mexican sand flies in a Latin America context, using the COI barcode and MALDI-TOF, including the correlation between males and females of a same species, uncovering a hidden diversity of species. Also, new COI barcode for some species are provided, as well as molecular records from areas where they were previously unknown. Different assemblages of MOTUs were obtained with each algorithm tested, but especially for species of the genera *Lutzomyia*, *Micropygomyia*, *Psathyromyia*, and *Pintomyia*, a higher genetic diversity was observed, which included cryptic species and potential new species or lineages for Mexico. Unlike other studies in which the GMYC model overestimated the amount of MOTUs (Rodrigues et al. 2024b), in this study GMYC and PTP were the most accurate models for species delimitation. The most important findings of the analysis are highlighted below, notwithstanding complementary studies are still necessary.

### Implementation of MALDI-TOF for Mexican Sand Flies Identification

In the New World MALDI-TOF MS result rapid and reliable tool for promising results to species identification, blood-meal preferences and/or pathogen detection of sand flies, in Brazil (Silva et al. 2024), Ecuador (Mathis et al. 2015), and French Guiana (Chavy et al. 2019). In this study, we analyzed the thorax, wing, and legs of each specimens, and for all species a reproducible species-specific protein spectrum was obtained, which agrees with previous studies that utilized the same structures (Dvorak et al. 2014, Arfuso et al. 2019).

A successful protein extraction for mass spectrometry analysis was a challenge. However, a distilled water for at least 24 h to eliminate excess ethanol in thorax, aid quality extraction for older samples. Yet, it is important to highlight that the rehydration process could introduce potential biases or artifacts in the reading of protein profiles, so complementary replicates would be useful to record variability for Mexican sand flies identification using MALDI-TOF technique.

A recommendation to perform the MALDI-TOF MS is dry–freeze (−80 °C) the specimens instead of using ethanol, especially if the specimens will be stored for a long time, because ethanol may induce protein precipitation, reducing their solubility and constraining the quality or mass spectrometry, which impedes a correct species identification of insects (Dvorak et al. 2014, Mathis et al. 2015, Arfuso et al. 2019, Chavy et al. 2019). However, when freezing in the field is no attainable, rinsing in distilled water is still a viable option.

Our findings suggest that MALDI-TOF MS is a useful tool for obtaining complementary information for taxonomic identification of sand flies as each species has a unique profile. The protein profiles obtained are the first for the species analyzed, contributing new information for identification of species of the genera *Bichromomyia*, *Micropygomyia*, *Psathyromyia*, *Psychodopygus*, *Trichopygomyia*, which already have previous information for other species (Chavy et al. 2019).

It is important to highlight that these findings are still preliminary, as we could only analyze specimens from single localities, so further studies on species with a broader geographic distribution are necessary to test this technique for a precise taxonomic identification of sand flies and also allow to discern cryptic diversity (Dvorak et al. 2014). Some studies suggest that MALDI-TOF could have a higher resolution than DNA barcoding, but this should still be evaluated as this tool has not been efficient to separate cryptic and/or closely related species in other Diptera (Chavy et al. 2019). Furthermore, the creation of a formal open-access library for mass spectrometry profiling is recommended as a complementary tool to morphological and molecular approaches (Chavy et al. 2019, Silva et al. 2024).

### Is *Lutzomyia cruciata* a Species Complex?

*Lutzomyia cruciata* has epidemiological importance for its role as a vector of *Leishmania* spp., being widely distributed in Mexico (Lozano-Sardaneta et al. 2024a). Previous studies focused on *Lu*. *cruciata* from the state of Chiapas, suggested that it consists of a species complex, considering its high genetic diversity (using cytochrome b gene) and morphometric variability (Pech-May et al. 2013, Mikery et al. 2019). In addition, it was recently recorded infected with Vesicular Stomatitis Virus (Zhou et al. 2024), therefore, its epidemiological importance has increased, and a correct species delimitation is pertinent. Using the COI gene, our results support that at least 2 MOTUs are distributed in Chiapas, which may represent cryptic species, with high genetic variability (7.4% to 9.3%), and *Fst* value near 0.8. One of this MOTUs was similar to a sequence from Colombia (Contreras-Gutiérrez et al. 2014), therefore complementary morphological and molecular studies in Chiapas, Central America, and Colombia should be pertinent to delimit these cryptic species and delimit their phylogeographic distribution.

On the contrary, the other MOTU also found in Chiapas, seems an endemic Mexican lineage widely distributed in the country, with a population structure that ranges between low and moderate values (*Fst* = 0.12 to 0.69), and an intraspecific variability of 3%, which results an acceptable variation for COI barcoding in sand flies (0% to 6%) (Contreras-Gutiérrez et al. 2014). Yet, the populations distributed in Nuevo León and Veracruz displayed the highest values of population structure in comparison to populations from Chiapas or Quintana Roo. The populations from NL and Ver might also represent cryptic species, but available molecular information is currently limited to confirm this hypothesis, however, the genetic variation observed should not be ignored in further studies.

*Lutzomyia cruciata* is an older taxon that could have diverged approximately at 67.14 Mya (Lozano-Sardaneta et al. 2023a), which could explain its high intraspecific diversity and phenotypic plasticity at the morphometric and exochorionic levels that allow a better adaptability to different abiotic conditions as a result of its evolutionary history in Mexico (Mikery et al. 2019, Lozano-Sardaneta et al. 2024b, 2025). A similar pattern has been recorded in *Phlebotomus ariasi* Tonnoir in France, which also recorded high phenotypic plasticity across its distribution, as a result of environmental pressures that make it a species more sensitive to extrinsic factors (Prudhomme et al. 2016). Other species of the same subgenus, *Tricholateralis*, for instance *Lutzomyia gomezi* (Nitzulescu), has been recorded with a high intraspecific variation of 6%, that initially was considered an acceptable variation for a species, but recent complementary information supports that this taxon includes cryptic species in Colombia (Contreras-Gutiérrez et al. 2014, Posada-López et al. 2023).

### Recognition of Cryptic Species for the Genus *Micropygomyia*

In this study, 3 species of genus *Micropygomyia* Series cayennensis were analyzed; one of the remarkable findings is that the genetic distances between these sand flies suggest that there are 5 potential cryptic species. Firstly, *Mi. cay. cayennensis* (Col I /Pan and Col II) clustered in 2 different genetic MOTUs with 4.5% of differences, this finding reinforces a previous study that suggest that these are a cryptic species distributed between Colombia and Panama (Posada-López et al. 2023). The speciation process as result of land fragmentation and geographic distance distribution could be a reason of these genetic differences; however, another possibility for this species could be related with endosymbiotic interactions that may also result in benefic associations that favor genetic isolation, for instance, *Mi. cay. cayennensis* has been recorded with 2 different *Cardinium* strains in Colombia, a bacteria that causes reproductive abnormalities in arthropods (Vivero-Gomez et al. 2021). A similar case was proposed for the reproductive bacterial *Wolbachia* and *Pa*. *shannoni* in Mexico, mainly in populations from Quintana Roo (Martínez-Burgos et al. 2023). Whether the association between endosymbionts and sand flies may also contribute to the speciation process by reproductive isolation is something that remains to be evaluated, but it may be a working hypothesis to explain why some sand fly species are morphologically similar but genetically different, especially when there is a sympatric distribution.

Series cayennensis included 8 subspecies, and the first described was *Mi. cay. cayennensis* from FG (Shimabukuro et al. 2017). At compared the genetic information of *Mi. cayennensis* from FG, high genetic diversity was detected (18%) regarding the *Mi. cay. cayennensis* from Colombia/Panama, suggesting that they are different species. Until now, *Mi. cay. cayennensis* from FG is the only subspecies recorded in this country, yet this subspecies has been recorded in at least 14 countries including Mexico (Shimabukuro et al. 2017). Furthermore, it would be advisable to carry out a comprehensive complementary morphological and molecular review to achieve a correct taxonomic placement of this subspecies, as *Mi. cay. cayennensis* could potentially be a species complex. Something similar occurs when the molecular information of *Mi. cay. maciasi* from Mexico was compared, with *Mi. cay. cayennensis* from FG (10%) and Col/Pan (16%), their genetic distance varying highly, which does not support that they are considered at the subspecies category, and a revision of the taxonomic status of these 8 subspecies would be pertinent since it is possible that other subspecies be raised at nominal species status.

Interesting in specimens identify as *Mi*. *durani* clustered in two different species with a relatively large genetic distance (13%), the molecular and morphological information confirmed that one cluster (sp. 1) belong to the nominal species *Mi*. *durani* which was collected in the states of Puebla and Oaxaca. But regarding to the other sequences identified as *Mi*. aff. *durani* (sp. 2) might be considered a putative new species distributed in sympatry with *Mi*. *durani* (sp. 1) in Oaxaca. Sympatric speciation has been recorded that could occur in sand flies that occupy the same geographic location, for instance *Sergentomyia bailyi* (Sinton), or *L. longipalpis*, as result of the independent selection of their ecological niches, within the same area as the parental group causing reproductive isolation (possibly also related with endosymbionts, see above) (Yogeswari and Srinivasan 2016). For now, it is not advisable to make a formal description of this new species because the males of both species are isomorphic, and the females share many morphological similarities, except for the arrangement of the pharyngeal spines, so a complementary study focused on the variations in the morphological characteristics in the specimens previously identified as *Mi*. *durani* in Mexico is recommended.

The misidentifications between species that share morphological similarities are common, and represent a limitation for classical taxonomy. For instance, species such as *Sciopemyia microps* (Mangabeira) from Brazil, *Nyssomyia fraihai* (Martins, Falcão & Silva) from Colombia, *Sergentomyia gemmea* Lewis & Jeffery and *Sergentomyia iyengari* (Sinton) from Thailand, or *Sergentomyia babu* (Annandale) and *S. bailyi* (Sinton) from India, has only been possible to be separated considering their large genetic differences (Kumar et al. 2012, Pinto et al. 2015, Posada-López et al. 2023, Soomro et al. 2025). Although the epidemiological relevance of species of the genus *Micropygomyia* is unknown in Mexico, the information obtained highlights that the Series cayennensis needs a deep taxonomic revision as several species have probably been misidentified due to their morphological similarity.

### Large Genetic Structure in the Genus *Psathyromyia*

The species *Pa*. *shannoni* is widely distributed in Mexico, with a great epidemiological importance as one of the main vectors of *Leishmania* (Lozano-Sardaneta et al. 2024a). This species, along with 5 other species, are considered part of the Shannoni species complex due to their high morphological similarities, but show a high genetic divergence (Pinto et al. 2015, Sábio et al. 2016, Lozano-Sardaneta et al. 2023a). In a previous study using information of sand fly *Pa*. *shannoni* from Quintana Roo, the distribution of at least two lineages in Mexico was recorded (Lozano-Sardaneta et al. 2023b), which agrees with our results. The MOTU named in this study QRI was previously recorded as similar to sequences from Colombia, while the MOTU QRII result unique for Mexico (Lozano-Sardaneta et al., 2023b). With this analysis, the presence of two cryptic species within the Shannoni species complex distributed in QR seems overwhelming, and results complementary to a recent wing geometric morphometric study, where significant differences in the wing size and shape of specimens distributed in this state were recorded (Lozano-Sardaneta et al. 2025). The presence of different lineages in sympatric distribution could be related to habitat fragmentation of natural areas by human activities in QR that favor recent speciation events, so further research on morphological and molecular differentiation of these cryptic diversity should be important as this species is one of the main vectors of *Leishmania* in this state (Martínez-Burgos et al. 2023). In addition to these two QR MOTUs, two others MOTUs (NL and Ver) split in the ML tree, all four have high genetic structure (*Fst* = 0.9), confirming that geographical barriers in Mexico (limiting Nearctic and Neotropical areas) hinder gene flow between *Pa*. *shannoni* populations throughout its wide distribution (Lozano-Sardaneta et al. 2024a). This molecular evidence represents an opportunity for future taxonomic research, since in addition to *Pa*. *shannoni sensu stricto*, there could be other species of the Shannoni species complex distributed in Mexico, for which thoracic morphological characters that separate species of this complex should be incorporated before DNA or protein extraction (Sábio et al. 2014, 2016).

Conversely, *Pa*. *texana* splits their sequences in two MOTUs with a genetic variation of 5.38%. This genetic difference is probably related to the populations analyzed being geographically distant, and is only part of the intraspecific variation accepted for sand flies (Contreras-Gutiérrez et al. 2014), as at this moment the molecular information is limited it may be better to include additional molecular evidence before confirming potential cryptic diversity in Mexico. On the contrary, molecular and genetic evidence showed that *Pa*. *carpenteri* from Caldas, Colombia, is a cryptic species because of its large genetic distance (7%, *Fst* = 0.9) regarding the sequences from Mexico, Panama, and Colombia (Sucre), while the specimens distributed in Mexico seem to belong to the same lineage that is distributed as far as Panama and Colombia (Sucre), with a genetic variation of 3% and a high genetic structure (*Fst* = 09). Because the populations analyzed are from very distant areas that could affect the genetic flow, at this moment, the hypothesis that they are the same lineage or MOTU seems more plausible, and is support by the algorithms ABGD, GYMC, and PTP that were the models considered better for our analysis, yet additional evidence is still necessary to clarify whether the Mexican specimens represent other cryptic species inside of this taxon.

### *Pintomyia* Species Hidden Genetic Variation

In Mexico, only the species *Pi*. *serrana*, *Pintomyia evansi* (Nuñez-Tovar), *Pi*. *ovallesi* and *Pintomyia itza* Ibáñez-Bernal, May-UC & Rebollar-Tellez, has been recorded at the moment (Shimabukuro et al. 2017). Our results showed that *Pi*. *ovallesi* has a high population structure across its distribution, and at least two different MOTUs were identified, one distributed between Quintana Roo and Tabasco states, and another in Chiapas, which turned out similar to specimens from Panama. For the first MOTU, which might be endemic to Mexico, a wing geometric morphometric analysis, did not record morphological differences between these specimens, supporting that all belong to the same species (Lozano-Sardaneta et al. 2025). But for the second MOTU, their genetic differentiation could be favored by its geographic distribution. The state of Chiapas is located in southeastern of Mexico, on the border with Guatemala, where three biogeographic provinces converge, with specific and contrasting abiotic characteristics (Morrone et al. 2002). Although further studies will still be necessary, the geographic location of Chiapas could favor genetic differentiation between some lineages of sand flies distributed in North America, with regard to those distributed in Central and South America, as a possible transition zone, as observed in *Lu*. *cruciata*. In Brazil, something similar was recorded for *Pintomyia monticola* (Costa Lima) and *Pintomyia misionensis* (Castro), which throughout their distribution can hide a high cryptic diversity at the molecular level, but at morphological level (including geometric and linear morphometrics studies) they do not present differences, which disregards a formal description for each new recorded MOTUs (Rodrigues et al. 2024b).

Additionally, molecular information of a male specimen collected from Oaxaca was recovered. Its morphology suggests that it belongs to a species similar to *Pi*. *serrana*, which was originally described from Brazil, but is distributed in 13 New World countries (Shimabukuro et al. 2017). Recently, COI barcode studies demonstrated that *Pi*. *serrana* holds cryptic diversity across its range in Brazil, suggesting that may constitute a species complex (Pinto et al. 2023, Rodrigues et al. 2024a). However, when comparing the sequences of *Pi*. *serrana* from Brazil with ours, a high genetic distance (20%) was observed, and there are no similar sequences in Genbank that allow us to confirm the identity of this specimen. The genus *Pintomyia*, includes several species of epidemiological importance in Latin America (Rodrigues et al. 2024b), therefore a comprehensive morphological and ecological revision is warranted for the correct placement of this specimen, as the molecular evidence supports that it may represent a putative new species from Mexico, with morphological similarities to species of the Series serrana (Pereira et al. 2019, Galati 2023).

Based on the results obtained it is worth highlighting that richness of Mexican sand flies may be underestimated. Therefore, discovery of hidden species diversity could have a positive impact on efforts to control and prevent disease transmission. The discovery of higher genetic diversity could be related to Mexico’s orographic complexity, of Nearctic and Neotropical affinity, which favors a high ecosystem diversity and endemism. This causes its phylogeographic patterns to differ from the fauna of Central and South America, especially for taxa with a wide geographic distribution, such as *Pi. serrana*, *Mi. cayennensis*, *Psathyromyia carpenteri*, *Ps. shannoni*, and *Lu. cruciata*. It should be noted that establishing a standard threshold of genetic variation to delimit taxa or recognize their natural history is not an easy task, as species go through different microevolutionary processes, generating inconsistencies between analyses using integrative taxonomic approach (Posada-López et al., 2023, Rodrigues et al., 2024b). Therefore, complementary morphological and molecular information is still required to assign a correct taxonomic status to several sand fly species and demonstrate their epidemiological relevance. In sand flies, cryptic diversity is frequently explained as a consequence of geographic isolation. However, in other insects (eg stick insects and leaf insects), it has been shown that closely related lineages or taxa can follow parallel evolutionary trajectories, leading to morphological convergence or phenotypic repeatability, as a consequence of adaptation to similar environmental challenges (Boisseau et al. 2025). While this has not been demonstrated for sand flies, other evolutionary processes such as morphological convergence or reproductive isolation caused by endosymbionts should be considered in future studies, as each taxon is likely to undergo speciation processes under different circumstances.

## Supplementary material

Supplementary material is available at *Journal of Insect Science* online.

*Conflicts of interest*. None declared.

ieaf070_suppl_Supplementary_Tables_S1-S3

ieaf070_suppl_Supplementary_Tables_S4

ieaf070_suppl_Supplementary_Tables_S5

ieaf070_suppl_Supplementary_Figures_S1

ieaf070_suppl_Supplementary_Figures_S2

## Data Availability

The data that supports the findings of this study are available in the supporting information of this article. The accession numbers for the GenBank are PV367443 to PV367482, and for BOLD systems are YOK001-25 to YOK040-25.
