# Supplementary material for: Appearance may be deceiving: Mexican sand flies (Diptera: Psychodidae: Phlebotominae) embrace a high diversity of cryptic species
Source: J Insect Sci. 2025 Jul 25;25(4):4. doi: 10.1093/jisesa/ieaf070 (PMC12290217; doi:10.1093/jisesa/ieaf070)
Supplement: ieaf070_suppl_Supplementary_Tables_S1-S3 [file ieaf070_suppl_supplementary_tables_s1-s3.doc]

**Appearance may be deceiving: Mexican sand flies (Diptera: Psychodidae: Phlebotominae) embrace a high diversity of cryptic species**

**Supplementary Table S1. List of sand fly species analyzed, including geographic data, GenBank and Bold numbers.**

| **Code** | **Genus** | **Specie** | **Sex** | **State** | **Municipally** | **Locality** | **Date** | **Latitude** | **Longitude** | **Altitude** | **GenBank number** | **Bold number** | **MALDI-TOF** |
| --- | --- | --- | --- | --- | --- | --- | --- | --- | --- | --- | --- | --- | --- |
| 1F | *Dampfomyia* | *deleoni* | female | Quintana Roo | Othon P. Blanco | Nicolás Bravo | 1-Sep-2021 | 18.45918 | -88.92902 | 104 | PV367443 | YOK001-25 | Yes |
| 4F | *Dampfomyia* | *deleoni* | female | Quintana Roo | Othón P. Blanco | Nicolás Bravo | 22-Dec-2021 | 18.45918 | -88.92902 | 104 | PV367444 | YOK002-25 | N/A |
| 12F | *Dampfomyia* | *deleoni* | female | Quintana Roo | Othón P. Blanco | Huay Pix | 12-Jul-2022 | 18.517701 | -88.423509 | 10 | PV367445 | YOK003-25 | N/A |
| 13F | *Psathyromyia* | *shannoni* | female | Quintana Roo | Othón P. Blanco | Huay Pix | 12-Jul-2022 | 18.517701 | -88.423509 | 10 | PV367446 | YOK004-25 | N/A |
| 14F | *Psathyromyia* | *shannoni* | male | Quintana Roo | Othón P. Blanco | La Unión | 28-Mar-2020 | 17.897222 | -88.880556 | 70 | PV367447 | YOK005-25 | N/A |
| 15F | *Psathyromyia* | *shannoni* | female | Quintana Roo | Bacalar | Los divorciados | 19-Oct-2020 | 19.076390 | -87.456389 | 40 | PV367448 | YOK006-25 | N/A |
| 16F | *Lutzomyia* | *cruciata* | female | Quintana Roo | Bacalar | Los divorciados | 19-Oct-2020 | 19.076390 | -87.456389 | 40 | PV367449 | YOK007-25 | N/A |
| 17F | *Dampfomyia* | *beltrani* | female | Quintana Roo | Puerto Morelos | Central Vallarta | 11-Apr-2020 | 20.863333 | -87.049167 | 5 | PV367450 | YOK008-25 | N/A |
| 18F | *Lutzomyia* | *cruciata* | female | Quintana Roo | Tulum | Francisco Hu May | 1-Jun-2021 | 20.3333 | -87.5667 | 20 | PV367451 | YOK009-25 | N/A |
| 22F | *Dampfomyia* | *deleoni* | female | Quintana Roo | Othón P. Blanco | Caobas | 1-Apr-2020 | 18.444167 | -89.104167 | 150 | PV367452 | YOK010-25 | N/A |
| 23F | *Pintomyia* | *ovallesi* | female | Quintana Roo | Othón P. Blanco | Nicolás Bravo | 1-Mar-2021 | 18.457778 | -88.927778 | 100 | PV367453 | YOK011-25 | N/A |
| 25F | *Pintomyia* | *ovallesi* | female | Tabasco | Cunduacán | J. M. Pino Suárez | 3-Aug-2009 | 18.149167 | -93.291667 | 10 | PV367454 | YOK012-25 | N/A |
| 26F | *Psathyromyia* | *shannoni* | female | Quintana Roo | Othón P. Blanco | Nicolás Bravo | 22-Dec-2021 | 18.45918 | -88.92902 | 104 | PV367455 | YOK013-25 | N/A |
| 41F | *Lutzomyia* | *cruciata* | female | Quintana Roo | Othón P. Blanco | Nicolás Bravo | 1-Jun-2021 | 18.457778 | -88.927778 | 100 | PV367456 | YOK014-25 | N/A |
| 43F | *Dampfomyia* | *deleoni* | male | Quintana Roo | Othón P. Blanco | Huay Pix | 20-Dec-2021 | 18.517701 | -88.423509 | 10 | PV367457 | YOK015-25 | N/A |
| 45F | *Psathyromyia* | *cratifer* | female | Quintana Roo | Othón P. Blanco | Nicolás Bravo | 22-Dec-2021 | 18.45918 | -88.92902 | 104 | PV367458 | YOK016-25 | N/A |
| 46F | *Pintomyia* | *ovallesi* | female | Quintana Roo | Othón P. Blanco | Nicolás Bravo | 22-Dec-2021 | 18.45918 | -88.92902 | 104 | PV367459 | YOK017-25 | N/A |
| 47F | *Pintomyia* | *ovallesi* | female | Quintana Roo | Othón P. Blanco | Nicolás Bravo | 22-Dec-2021 | 18.45918 | -88.92902 | 104 | PV367460 | YOK018-25 | N/A |
| 55F | *Pintomyia* | *ovallesi* | female | Quintana Roo | Othón P. Blanco | Nicolás Bravo | 22-Dec-2021 | 18.45918 | -88.92902 | 104 | PV367461 | YOK019-25 | N/A |
| 60F | *Dampfomyia* | *deleoni* | female | Quintana Roo | Othón P. Blanco | Huay Pix | 20-Dec-2021 | 18.517701 | -88.423509 | 10 | PV367462 | YOK020-25 | N/A |
| 61F | *Dampfomyia* | *beltrani* | female | Quintana Roo | Felipe Carrillo Puerto | Dzula | 5-Sep-2018 | 18.544611 | -88.35333 | 40 | PV367463 | YOK021-25 | Yes |
| 78F | *Lutzomyia* | *cruciata* | female | Nuevo León | Santiago | Las adjuntas | 4-Aug-2023 | 25.30093 | -100.1381 | 731 | PV367464 | YOK022-25 | N/A |
| 79F | *Psathyromyia* | *shannoni* | female | Nuevo León | Santiago | Las adjuntas | 3-Aug-2023 | 25.30093 | -100.1381 | 731 | PV367465 | YOK023-25 | N/A |
| 80F | *Psathyromyia* | *texana* | female | Nuevo León | Santiago | Las adjuntas | 3-Aug-2023 | 25.30093 | -100.1381 | 731 | PV367466 | YOK024-25 | N/A |
| 82F | *Psathyromyia* | *shannoni* | male | Nuevo León | Santiago | Las adjuntas | 4-Aug-2023 | 25.30093 | -100.1381 | 731 | PV367467 | YOK025-25 | N/A |
| 100F | *Psathyromyia* | *shannoni* | female | Oaxaca | Santa María Chimalapan | Santa Inés Chimalapa | 30-Sep-2001 | 16.772222 | -94.804444 | 283 | PV367468 | YOK026-25 | N/A |
| 102F | *Micropygomyia* | *cayennensis maciasi* | female | Morelos | Puente de Ixtla | El Estudiante | 1-Apr-2011 | 18.577778 | -99.295278 | 870 | PV367469 | YOK027-25 | N/A |
| 107F | *Psathyromyia* | *texana* | female | Morelos | San José | Vista Hermosa | 15-Dec-2012 | 18.653611 | -99.265556 | 990 | PV367470 | YOK028-25 | N/A |
| 110F | *Psathyromyia* | *texana* | female | Morelos | San José | Vista Hermosa | 14-Nov-2013 | 18.653611 | -99.265556 | 990 | PV367471 | YOK029-25 | N/A |
| 114F7 sp1 | *Micropygomyia* | *durani* | female | Oaxaca | Santo Domingo de Morelos | Las cuevas | 3-Dec-2019 | 15.834444 | -96.666944 | 155 | PV367472 | YOK030-25 | N/A |
| 115F/sp2 | *Micropygomyia* | *durani* | male | Oaxaca | Santo Domingo de Morelos | Las cuevas | 3-Dec-2019 | 15.834444 | -96.666944 | 155 | PV367473 | YOK031-25 | N/A |
| 116F/sp1 | *Micropygomyia* | *durani* | female | Oaxaca | Santo Domingo de Morelos | Las cuevas | 3-Dec-2019 | 15.834444 | -96.666944 | 155 | PV367474 | YOK032-25 | N/A |
| 117F/sp2 | *Micropygomyia* | *durani* | female | Oaxaca | Santo Domingo de Morelos | Las cuevas | 3-Dec-2019 | 15.834444 | -96.666944 | 155 | PV367475 | YOK033-25 | N/A |
| 119F/sp1 | *Micropygomyia* | *durani* | male | Oaxaca | Santa María Tonameca | Cerro Gordo | 2-Dec-2019 | 15.7930 | -96.5938 | 142 | PV367476 | YOK034-25 | N/A |
| 132F/sp1 | *Micropygomyia* | *durani* | male | Oaxaca | Santo Domingo de Morelos | Las cuevas | 3-Dec-2019 | 15.834444 | -96.666944 | 155 | PV367477 | YOK035-25 | N/A |
| 143F | *Micropygomyia* | *cayennensis maciasi* | female | Morelos | Puente de Ixtla | El Estudiante | 1-Apr-2011 | 18.577778 | -99.295278 | 870 | PV367478 | YOK036-25 | N/A |
| 15Y | *Psathyromyia* | *maya* | female | Quintana Roo | Felipe Carrillo Puerto | Dzula | 5-Sep-2018 | 18.544611 | -88.35333 | 40 | PV367479 | YOK037-25 | N/A |
| 6F/sp1 | *Micropygomyia* | *durani* | female | Puebla | Zapotitlán | Jardín Botánico Helia Bravo | 11-Jun-2024 | 18.3262 | -97.4508 | 1426 | PV367480 | YOK038-25 | N/A |
| 7F/sp1 | *Micropygomyia* | *durani* | male | Puebla | Zapotitlán | Jardín Botánico Helia Bravo | 11-Jun-2024 | 18.3262 | -97.4508 | 1426 | PV367481 | YOK039-25 | N/A |
| 8F | *Pintomyia* | sp. | male | Oaxaca | Villa Tamazulapam del Progreso | Ojo de Agua Grande | 18-Jun-2024 | 17.06893 | -97.55815 | 2024 | PV367482 | YOK040-25 | N/A |
|  | *Bichromomyia* | *olmeca* | female | Quintana Roo | Huay Pix |  | 20-Dec-2021 | 18.517701 | -88.423509 | 10 | N/A | N/A | Yes |
|  | *Bichromomyia* | *olmeca* | female | Quintana Roo | Huay Pix |  | 20-Dec-2021 | 18.517701 | -88.423509 | 10 | N/A | N/A | Yes |
|  | *Bichromomyia* | *olmeca* | female | Quintana Roo | Huay Pix |  | 20-Dec-2021 | 18.517701 | -88.423509 | 10 | N/A | N/A | Yes |
|  | *Brumptomyia* | *mesai* | female | Quintana Roo | Bacalar | Limones | 19-Mar-2014 | 18.9961 | -88.1575 | 19 | N/A | N/A | Yes |
|  | *Brumptomyia* | *mesai* | female | Quintana Roo | Bacalar | Limones | 19-Mar-2014 | 18.9961 | -88.1575 | 19 | N/A | N/A | Yes |
|  | *Brumptomyia* | *mesai* | female | Quintana Roo | Bacalar | Limones | 19-Mar-2014 | 18.9961 | -88.1575 | 19 | N/A | N/A | Yes |
|  | *Brumptomyia* | *mesai* | female | Quintana Roo | Bacalar | Limones | 19-Mar-2014 | 18.9961 | -88.1575 | 19 | N/A | N/A | Yes |
|  | *Brumptomyia* | *mesai* | female | Quintana Roo | Bacalar | Limones | 19-Mar-2014 | 18.9961 | -88.1575 | 19 | N/A | N/A | Yes |
|  | *Brumptomyia* | *mesai* | female | Quintana Roo | Bacalar | Limones | 19-Mar-2014 | 18.9961 | -88.1575 | 19 | N/A | N/A | Yes |
|  | *Brumptomyia* | *mesai* | female | Quintana Roo | Bacalar | Limones | 19-Mar-2014 | 18.9961 | -88.1575 | 19 | N/A | N/A | Yes |
|  | *Dampfomyia* | *deleoni* | female | Quintana Roo | Othon P. Blanco | Nicolás Bravo | 1-Sep-2021 | 18.45918 | -88.92902 | 104 | N/A | N/A | Yes |
|  | *Dampfomyia* | *deleoni* | female | Quintana Roo | Othón P. Blanco | Nicolás Bravo | 1-Sep-2021 | 18.45918 | -88.92902 | 104 | N/A | N/A | Yes |
|  | *Dampfomyia* | *deleoni* | female | Quintana Roo | Felipe Carrillo Puerto | Andrés Quintana Roo | 16-May-2019 | 19.161111 | -88.102778 | 30 | N/A | N/A | Yes |
|  | *Dampfomyia* | *deleoni* | female | Quintana roo | Felipe Carrillo Puerto | Andrés Quintana Roo | 16-May-2019 | 19.161111 | -88.102778 | 30 | N/A | N/A | Yes |
|  | *Pintomyia* | *ovallesi* | female | Quintana Roo | Bacalar | Limones | 25-Jan-2014 | 18.9961 | -88.1575 | 19 | N/A | N/A | Yes |
|  | *Pintomyia* | *ovallesi* | female | Quintana Roo | Bacalar | Limones | 25-Jan-2014 | 18.9961 | -88.1575 | 19 | N/A | N/A | Yes |
|  | *Pintomyia* | *ovallesi* | female | Quintana Roo | Bacalar | Limones | 25-Jan-2014 | 18.9961 | -88.1575 | 19 | N/A | N/A | Yes |
|  | *Pintomyia* | *ovallesi* | female | Quintana Roo | Bacalar | Limones | 25-Jan-2014 | 18.9961 | -88.1575 | 19 | N/A | N/A | Yes |
|  | *Pintomyia* | *ovallesi* | female | Quintana Roo | Bacalar | Limones | 25-Jan-2014 | 18.9961 | -88.1575 | 19 | N/A | N/A | Yes |
|  | *Pintomyia* | *ovallesi* | female | Quintana Roo | Bacalar | Limones | 25-Jan-2014 | 18.9961 | -88.1575 | 19 | N/A | N/A | Yes |
|  | *Pintomyia* | *ovallesi* | female | Quintana Roo | Bacalar | Limones | 25-Jan-2014 | 18.9961 | -88.1575 | 19 | N/A | N/A | Yes |
|  | *Pintomyia* | *ovallesi* | female | Quintana Roo | Bacalar | Limones | 25-Jan-2014 | 18.9961 | -88.1575 | 19 | N/A | N/A | Yes |
|  | *Lutzomyia* | *cruciata* | female | Quintana Roo | Bacalar | Limones | 25-Jan-2014 | 18.9961 | -88.1575 | 19 | N/A | N/A | Yes |
|  | *Lutzomyia* | *cruciata* | female | Quintana Roo | Felipe Carrillo Puerto | Andrés Quintana Roo | 16-May-2019 | 19.161111 | -88.102778 | 30 | N/A | N/A | Yes |
|  | *Lutzomyia* | *cruciata* | female | Quintana Roo | Felipe Carrillo Puerto | Andrés Quintana Roo | 16-May-2019 | 19.161111 | -88.102778 | 30 | N/A | N/A | Yes |
|  | *Lutzomyia* | *cruciata* | female | Quintana Roo | Felipe Carrillo Puerto | Andrés Quintana Roo | 16-May-2019 | 19.161111 | -88.102778 | 30 | N/A | N/A | Yes |
|  | *Lutzomyia* | *cruciata* | female | Quintana Roo | Felipe Carrillo Puerto | Andrés Quintana Roo | 16-May-2019 | 19.161111 | -88.102778 | 30 | N/A | N/A | Yes |
|  | *Lutzomyia* | *cruciata* | female | Quintana Roo | Felipe Carrillo Puerto | Andrés Quintana Roo | 16-May-2019 | 19.161111 | -88.102778 | 30 | N/A | N/A | Yes |
|  | *Psathyromyia* | *shannoni* | female | Quintana Roo | Felipe Carrillo Puerto | Andrés Quintana Roo | 16-May-2019 | 19.161111 | -88.102778 | 30 | N/A | N/A | Yes |
|  | *Psathyromyia* | *shannoni* | female | Quintana Roo | Felipe Carrillo Puerto | Andrés Quintana Roo | 16-May-2019 | 19.161111 | -88.102778 | 30 | N/A | N/A | Yes |
|  | *Psathyromyia* | *shannoni* | female | Quintana Roo | Felipe Carrillo Puerto | Andrés Quintana Roo | 16-May-2019 | 19.161111 | -88.102778 | 30 | N/A | N/A | Yes |
|  | *Psathyromyia* | *shannoni* | female | Quintana Roo | Felipe Carrillo Puerto | Andrés Quintana Roo | 16-May-2019 | 19.161111 | -88.102778 | 30 | N/A | N/A | Yes |
|  | *Psathyromyia* | *shannoni* | male | Quintana Roo | Felipe Carrillo Puerto | Andrés Quintana Roo | 16-May-2019 | 19.161111 | -88.102778 | 30 | N/A | N/A | Yes |
|  | *Psathyromyia* | *shannoni* | male | Quintana Roo | Felipe Carrillo Puerto | Andrés Quintana Roo | 16-May-2019 | 19.161111 | -88.102778 | 30 | N/A | N/A | Yes |
|  | *Psathyromyia* | *shannoni* | male | Quintana Roo | Felipe Carrillo Puerto | Andrés Quintana Roo | 16-May-2019 | 19.161111 | -88.102778 | 30 | N/A | N/A | Yes |
|  | *Psathyromyia* | *shannoni* | male | Quintana Roo | Felipe Carrillo Puerto | Andrés Quintana Roo | 16-May-2019 | 19.161111 | -88.102778 | 30 | N/A | N/A | Yes |
|  | *Tricopygomyia* | *triramula* | female | Oaxaca | Santa Inés Chimalapa |  | 31-Sep-2001 | 16.772222 | -94.804444 | 283 | N/A | N/A | Yes |
|  | *Tricopygomyia* | *triramula* | female | Oaxaca | Santa Inés Chimalapa |  | 31-Sep-2001 | 16.772222 | -94.804444 | 283 | N/A | N/A | Yes |
|  | *Micropygomyia* | *cayennensis* | male | Tabasco | Cunduacán | J. M. Pino Suárez | 3-Aug-2009 | 18.149167 | -93.291667 | 10 | N/A | N/A | Yes |
|  | *Micropygomyia* | *chiapanensis* | female | Veracruz | Ignacio Llave |  | 7-Feb-2001 | 18.66177 | -95.97212 | 7 | N/A | N/A | Yes |
|  | *Micropygomyia* | *chiapanensis* | female | Veracruz | Pozo de Arena |  | 3-Feb-2001 | 18.625 | -95.987222 | 10 | N/A | N/A | Yes |
|  | *Micropygomyia* | *durani* | male | Puebla | Zapotitlán | Jardín Botánico Helia Bravo | 11-Jun-2024 | 18.3262 | -97.4508 | 1426 | N/A | N/A | Yes |
|  | *Micropygomyia* | *durani* | male | Puebla | Zapotitlán | Jardín Botánico Helia Bravo | 11-Jun-2024 | 18.3262 | -97.4508 | 1426 | N/A | N/A | Yes |
|  | *Micropygomyia* | *durani* | female | Puebla | Zapotitlán | Jardín Botánico Helia Bravo | 11-Jun-2024 | 18.3262 | -97.4508 | 1426 | N/A | N/A | Yes |
|  | *Micropygomyia* | *durani* | male | Puebla | Zapotitlán | Jardín Botánico Helia Bravo | 11-Jun-2024 | 18.3262 | -97.4508 | 1426 | N/A | N/A | Yes |
|  | *Lutzomyia* | *longipalpis* | male | Oaxaca | Santo Domingo de Morelos | Cerro Campana | 21-Aug-1998 | 15.855 | -96.642222 | 420 | N/A | N/A | Yes |
|  | *Lutzomyia* | *longipalpis* | male | Oaxaca | Santo Domingo de Morelos | Cerro Campana | 21-Aug-1998 | 15.855 | -96.642222 | 420 | N/A | N/A | Yes |
|  | *Psychodopygus* | *panamensis* | male | Quintana Roo | Felipe Carrillo Puerto | Noh Bec | 16-Dec-2021 | 19.143056 | -88.169167 | 30 | N/A | N/A | Yes |
|  | *Psychodopygus* | *panamensis* | male | Quintana Roo | Felipe Carrillo Puerto | Noh Bec | 16-Dec-2021 | 19.143056 | -88.169167 | 30 | N/A | N/A | Yes |

**Supplementary Table S2**. List of sand fly sequences from GenBank downloaded to perform species delimitation.

| **Genbank Number** | **Genus** | **Specie** | **Subspecie** | **Country** | **Locality** |
| --- | --- | --- | --- | --- | --- |
| MT338247 | *Micropygomyia* | *cayennensis* | *cayennensis* | Colombia | Puerto Libertador, La Bonga |
| JN845561 | *Micropygomyia* | *cayennensis* | *cayennensis* | Colombia | Antioquia Santa Fé |
| OP964247 | *Micropygomyia* | *cayennensis* | *cayennensis* | Panama | Capira Ollas arriba |
| MT338250 | *Micropygomyia* | *cayennensis* | *cayennensis* | Colombia | Puerto Libertador, Pica Pica |
| GU909475 | *Micropygomyia* | *cayennensis* | *cayennensis* | Colombia | Sucre, Sincelejo |
| GU909473 | *Micropygomyia* | *cayennensis* | *cayennensis* | Colombia | Sucre,Coloso |
| KR907863 | *Micropygomyia* | *cayennensis* | *cayennensis* | Colombia |  |
| OP964248 | *Micropygomyia* | *cayennensis* | *cayennensis* | Colombia | Sucre, Ovejas |
| MT338249 | *Micropygomyia* | *cayennensis* | *cayennensis* | Colombia | Valencia-Zaino |
| OP964245 | *Micropygomyia* | *cayennensis* | *cayennensis* | Colombia | Sucre, Ovejas |
| MT338248 | *Micropygomyia* | *cayennensis* | *cayennensis* | Colombia | Montelibano-Puerto Anchica |
| GU909472 | *Micropygomyia* | *cayennensis* | *cayennensis* | Colombia | Sucre, Sincelejo |
| KX356032 | *Micropygomyia* | *cayennensis* |  | French Guiana | genoma |
| MK744153 | *Lutzomyia* | *cruciata* |  | Mexico | Veracruz, Los Tuxtlas |
| MK744152 | *Lutzomyia* | *cruciata* |  | Mexico | Veracruz, Los Tuxtlas |
| MK744154 | *Lutzomyia* | *cruciata* |  | Mexico | Veracruz, Los Tuxtlas |
| MK744155 | *Lutzomyia* | *cruciata* |  | Mexico | Veracruz, Los Tuxtlas |
| MK744156 | *Lutzomyia* | *cruciata* |  | Mexico | Veracruz, Los Tuxtlas |
| MK744151 | *Lutzomyia* | *cruciata* |  | Mexico | Veracruz, Los Tuxtlas |
| MK851248 | *Lutzomyia* | *cruciata* |  | Mexico | Quintana Roo, Chetumal Othon P. Blanco |
| MK851247 | *Lutzomyia* | *cruciata* |  | Mexico | Quintana Roo, Candelaria |
| OQ325337 | *Lutzomyia* | *cruciata* |  | Mexico | Chiapas |
| OP784399 | *Lutzomyia* | *cruciata* |  | Mexico | Chiapas, San Antonio Buenavista |
| OP784400 | *Lutzomyia* | *cruciata* |  | Mexico | Chiapas, Guadalupe Miramar |
| OP784401 | *Lutzomyia* | *cruciata* |  | Mexico | Quintana Roo, Noh Bec |
| OP784402 | *Lutzomyia* | *cruciata* |  | Mexico | Quintana Roo, Noh Bec |
| OR608115 | *Lutzomyia* | *cruciata* |  | Mexico | Nuevo León, Monterrey |
| OR608116 | *Lutzomyia* | *cruciata* |  | Mexico | Nuevo León, Monterrey |
| OP781331 | *Dampfomyia* | *beltrani* |  | Mexico | Quintana Roo |
| MK851246 | *Dampfomyia* | *beltrani* |  | Mexico | Quintana Roo |
| OP781332 | *Dampfomyia* | *beltrani* |  | Mexico | Quintana Roo |
| MK851245 | *Dampfomyia* | *beltrani* |  | Mexico | Quintana Roo |
| MK851251 | *Dampfomyia* | *deleoni* |  | Mexico | Quintana Roo, Candelaria |
| OP784406 | *Dampfomyia* | *deleoni* |  | Mexico | Quintana Roo, Noh Bec |
| OP784405 | *Dampfomyia* | *deleoni* |  | Mexico | Chiapas, Loma Bonita |
| MK851253 | *Dampfomyia* | *deleoni* |  | Mexico | Quintana Roo, Candelaria |
| MK851252 | *Dampfomyia* | *deleoni* |  | Mexico | Quintana Roo, Candelaria |
| MK851250 | *Dampfomyia* | *deleoni* |  | Mexico | Quintana Roo, Candelaria |
| MK851249 | *Dampfomyia* | *deleoni* |  | Mexico | Quintana Roo, Candelaria |
| OP784404 | *Pintomyia* | *ovallesi* |  | Mexico | Chiapas, Loma Bonita |
| OP784403 | *Pintomyia* | *ovallesi* |  | Mexico | Chiapas, San Antonio Buenavista |
| MN257603 | *Pintomyia* | *ovallesi* |  | Panama |  |
| GU001745 | *Pintomyia* | *ovallesi* |  | Panama | Barro Colorado Island |
| GU001746 | *Pintomyia* | *ovallesi* |  | Panama | Barro Colorado Island |
| GU001744 | *Pintomyia* | *ovallesi* |  | Panama | Barro Colorado Island |
| OP784408 | *Nyssomyia* | *ylephiethor* |  | Mexico | Chiapas, Guadalupe Miramar |
| OP784409 | *Nyssomyia* | *ylephiethor* |  | Mexico | Chiapas, Guadalupe Miramar |
| OP784411 | *Nyssomyia* | *ylephiethor* |  | Mexico | Chiapas, Guadalupe Miramar |
| KC755394 | *Psathtromyia* | *shannoni* |  | Mexico | Quintana Roo |
| MH035798 | *Psathtromyia* | *shannoni* |  | Mexico | Quintana Roo, Betania |
| MK851277 | *Psathtromyia* | *shannoni* |  | Mexico | Quintana Roo, Chetumal Othon P. Blanco |
| KC755397 | *Psathtromyia* | *shannoni* |  | Mexico | Quintana Roo |
| KC755396 | *Psathtromyia* | *shannoni* |  | Mexico | Quintana Roo |
| OP784412 | *Psathtromyia* | *shannoni* |  | Mexico | Chiapas, San Antonio Buenavista |
| OP784414 | *Psathtromyia* | *shannoni* |  | Mexico | Quintana Roo, Noh Bec |
| OP784413 | *Psathtromyia* | *shannoni* |  | Mexico | Quintana Roo, Noh Bec |
| MK744146 | *Psathtromyia* | *shannoni* |  | Mexico | Veracruz, Los Tuxtlas |
| MK744145 | *Psathtromyia* | *shannoni* |  | Mexico | Veracruz, Los Tuxtlas |
| OR608118 | *Psathtromyia* | *shannoni* |  | Mexico | Nuevo León, Monterrey |
| OR608117 | *Psathtromyia* | *shannoni* |  | Mexico | Nuevo León, Monterrey |
| MK851281 | *Psathtromyia* | *shannoni* |  | Mexico | Quintana Roo, Chetumal Othon P. Blanco |
| MK851279 | *Psathtromyia* | *shannoni* |  | Mexico | Quintana Roo, Chetumal Othon P. Blanco |
| MK851280 | *Psathtromyia* | *shannoni* |  | Mexico | Quintana Roo, Chetumal Othon P. Blanco |
| KC755395 | *Psathtromyia* | *shannoni* |  | Mexico | Quintana Roo |
| KC755396 | *Psathtromyia* | *shannoni* |  | Mexico | Quintana Roo, Chetumal Othon P. Blanco |
| MK851278 | *Psathtromyia* | *shannoni* |  | Mexico | Quintana Roo, Chetumal Othon P. Blanco |
| MK851276 | *Psathtromyia* | *shannoni* |  | Mexico | Quintana Roo, Chetumal Othon P. Blanco |
| MK851282 | *Psathtromyia* | *shannoni* |  | Mexico | Quintana Roo, Chetumal Othon P. Blanco |
| MK851285 | *Psathtromyia* | *shannoni* |  | Mexico | Quintana Roo, Chetumal Othon P. Blanco |
| MK851275 | *Psathtromyia* | *shannoni* |  | Mexico | Quintana Roo, Chetumal Othon P. Blanco |
| MK851284 | *Psathtromyia* | *shannoni* |  | Mexico | Quintana Roo, Chetumal Othon P. Blanco |
| MK851283 | *Psathtromyia* | *shannoni* |  | Mexico | Quintana Roo, Chetumal Othon P. Blanco |
| MK744137 | *Psychodopygus* | *panamensis* |  | Mexico | Veracruz, Los Tuxtlas |
| MK744136 | *Psychodopygus* | *panamensis* |  | Mexico | Veracruz, Los Tuxtlas |
| OP784417 | *Psychodopygus* | *panamensis* |  | Mexico | Quintana Roo, Noh Bec |
| OP784416 | *Psychodopygus* | *panamensis* |  | Mexico | Chiapas, Guadalupe Miramar |
| OP784415 | *Psychodopygus* | *panamensis* |  | Mexico | Chiapas, Guadalupe Miramar |
| KC921236 | *Psathtromyia* | *carpenteri* |  | Colombia | Caldas |
| KC921235 | *Psathtromyia* | *carpenteri* |  | Colombia | Caldas |
| OP964327 | *Psathtromyia* | *carpenteri* |  | Colombia | Colombia: Caldas, Norcasia |
| GU909444 | *Psathtromyia* | *carpenteri* |  | Colombia | Sucre, Coloso |
| GU909445 | *Psathtromyia* | *carpenteri* |  | Colombia | Sucre, Coloso |
| GU001730 | *Psathtromyia* | *carpenteri* |  | Panama | Barro Colorado Island |
| GU001729 | *Psathtromyia* | *carpenteri* |  | Panama | Barro Colorado Island |
| GU001731 | *Psathtromyia* | *carpenteri* |  | Panama | Barro Colorado Island |
| OP784390 | *Psathtromyia* | *carpenteri* |  | Mexico | Quintana Roo, Noh Bec |
| OQ325338 | *Psathtromyia* | *carpenteri* |  | Mexico | Chiapas |
| MK744138 | *Psathtromyia* | *carpenteri* |  | Mexico | Veracruz, Los Tuxtlas |
| MK744139 | *Psathtromyia* | *carpenteri* |  | Mexico | Veracruz, Los Tuxtlas |
| MK744140 | *Psathtromyia* | *carpenteri* |  | Mexico | Veracruz, Los Tuxtlas |
| MK744141 | *Psathtromyia* | *carpenteri* |  | Mexico | Veracruz, Los Tuxtlas |
| MK744142 | *Psathtromyia* | *carpenteri* |  | Mexico | Veracruz, Los Tuxtlas |
| MK744143 | *Psathtromyia* | *carpenteri* |  | Mexico | Veracruz, Los Tuxtlas |
| MK744144 | *Psathtromyia* | *carpenteri* |  | Mexico | Veracruz, Los Tuxtlas |
| MK851243 | *Brumptomyia* | *mesai* |  | Mexico | Quintana Roo, Chetumal Othón P. Blanco |
| MK851242 | *Brumptomyia* | *mesai* |  | Mexico | Quintana Roo, Chetumal Othón P. Blanco |
| MK851244 | *Brumptomyia* | *mesai* |  | Mexico | Quintana Roo, Chetumal Othón P. Blanco |
| OP784396 | *Brumptomyia* | *mesai* |  | Mexico | Quintana Roo, Noh Bec |
| OP784394 | *Brumptomyia* | *mesai* |  | Mexico | Quintana Roo, Noh Bec |
| OP784395 | *Brumptomyia* | *mesai* |  | Mexico | Quintana Roo, Noh Bec |
| MK744150 | *Brumptomyia* | *mesai* |  | Mexico | Veracruz, Los Tuxtlas |

**Supplementary Table S3. Genetic distances (Kimura 2-parameters) among 20 sand fly species with distribution in Mexico.**

| **Species (State or Country)** | **Intraspecific distances** | **1** | **2** | **3** | **4** | **5** | **6** | **7** | **8** | **9** | **10** | **11** | **12** | **13** | **14** | **15** | **16** | **17** | **18** | **19** | **20** |
| --- | --- | --- | --- | --- | --- | --- | --- | --- | --- | --- | --- | --- | --- | --- | --- | --- | --- | --- | --- | --- | --- |
| (1) *Micropygomyia durani* sp.1 (Oax/Pue) | 0.48 - 2.7 |  |  |  |  |  |  |  |  |  |  |  |  |  |  |  |  |  |  |  |  |
| (2) *Micropygomyia durani* sp.2 (Oax) | 0.97 | 13.16 |  |  |  |  |  |  |  |  |  |  |  |  |  |  |  |  |  |  |  |
| (3) *Pintomyia* sp. (Oax) | - | 20.04 | 20.06 |  |  |  |  |  |  |  |  |  |  |  |  |  |  |  |  |  |  |
| (4) *Micropygomyia cayennensis cayennensis*  (Col/Pan) | 0-2.45 | 18.26 | 19.18 | 22.19 |  |  |  |  |  |  |  |  |  |  |  |  |  |  |  |  |  |
| (5) *Micropygomyia cayennensis* (FG) | - | 16.48 | 19.69 | 19.19 | 18.38 |  |  |  |  |  |  |  |  |  |  |  |  |  |  |  |  |
| (6) *Micropygomyia cayennensis maciasi*  (Mor) | 0.16 | 13.84 | 17.63 | 19.41 | 16.55 | 10.20 |  |  |  |  |  |  |  |  |  |  |  |  |  |  |  |
| (7) *Psathyromyia cratifer* (NL/QR) | 0.64 - 1.25 | 18.11 | 16.82 | 20.79 | 19.28 | 19.84 | 19.06 |  |  |  |  |  |  |  |  |  |  |  |  |  |  |
| (8) *Psathyromyia texana* (NL/Mor/QR/USA) | 1.30 - 2.7 | 16.05 | 16.19 | 20.01 | 18.86 | 16.83 | 14.28 | 16.23 |  |  |  |  |  |  |  |  |  |  |  |  |  |
| (9) *Psathyromyia shannoni* (NL/QR/Ch/Ox/Ver) | 0 - 3.01 | 18.99 | 16.93 | 21.59 | 20.41 | 19.85 | 21.19 | 15.16 | 16.73 |  |  |  |  |  |  |  |  |  |  |  |  |
| (10) *Lutzomyia cruciata* (NL/Ch/QR/Ver/Col) | 0.053 - 3.50 | 17.18 | 19.59 | 19.68 | 17.66 | 15.72 | 15.52 | 18.09 | 18.26 | 18.08 |  |  |  |  |  |  |  |  |  |  |  |
| (11) *Psathyromyia carpenteri*  (Ver/Qr/Chi/Col/Pan) | 0 - 3.8 | 16.90 | 18.10 | 20.47 | 16.58 | 17.12 | 15.25 | 14.96 | 9.52 | 17.16 | 18.44 |  |  |  |  |  |  |  |  |  |  |
| (12) *Psychodopygus panamensis* (QR/Ch/Ver) | 0.16 - 1.08 | 15.22 | 18.18 | 22.93 | 17.80 | 19.50 | 15.19 | 18.49 | 14.69 | 17.15 | 16.45 | 14.98 |  |  |  |  |  |  |  |  |  |
| (13) *Dampfomyia deleoni* (QR/Ch) | 0 - 0.11 | 18.03 | 20.68 | 19.45 | 19.79 | 16.94 | 16.94 | 18.72 | 17.71 | 19.49 | 18.12 | 18.78 | 17.09 |  |  |  |  |  |  |  |  |
| (14) *Pintomyia ovallesi* (QR/Tab/Chi/Pan) | 0 - 3.1 | 19.60 | 18.27 | 19.47 | 19.38 | 18.94 | 19.84 | 18.60 | 19.46 | 18.28 | 18.11 | 19.99 | 20.59 | 20.59 |  |  |  |  |  |  |  |
| (15) *Psathyromyia maya* (QR) | 0.98 | 17.54 | 16.68 | 20.64 | 19.45 | 19.99 | 18.89 | 20.44 | 18.11 | 20.65 | 18.14 | 19.49 | 20.15 | 19.07 | 21.63 |  |  |  |  |  |  |
| (16) *Dampfomyia beltrani* (QR) | 0.896 | 21.33 | 20.84 | 22.16 | 23.70 | 19.57 | 21.79 | 20.19 | 22.84 | 23.01 | 20.64 | 23.59 | 22.70 | 16.15 | 23.11 | 20.93 |  |  |  |  |  |
| (17) *Brumptomyia mesai* (QR/Ver) | 2.25 | 19.71 | 17.95 | 20.77 | 20.33 | 19.30 | 21.16 | 18.31 | 17.05 | 22.05 | 18.79 | 17.58 | 19.55 | 19.09 | 20.36 | 20.67 | 21.95 |  |  |  |  |
| (18) *Brumptomyia hamata* (Ver) | - | 18.78 | 17.09 | 21.71 | 19.73 | 20.12 | 21.43 | 18.86 | 17.57 | 21.68 | 19.35 | 16.90 | 19.26 | 18.93 | 20.54 | 20.82 | 20.21 | 3.94 |  |  |  |
| (19) *Bichromomyia olmeca* (QR/Tab/Ch) | 0.85 | 15.34 | 16.85 | 18.05 | 19.79 | 17.00 | 14.83 | 19.24 | 17.95 | 18.60 | 16.75 | 17.45 | 14.73 | 16.56 | 17.71 | 19.37 | 21.26 | 18.70 | 19.48 |  |  |
| (20) *Nyssomyia ylephiletor* (Chi) | 0.215 | 17.36 | 16.82 | 20.52 | 16.09 | 19.15 | 18.13 | 17.31 | 15.69 | 14.97 | 18.85 | 16.75 | 15.83 | 16.97 | 16.29 | 18.54 | 22.31 | 19.81 | 19.59 | 15.43 |  |

**States from Mexico: QR=Quintana Roo, Chi=Chiapas, Tab=Tabasco, Oax=Oaxaca, Mor=Morelos, Ver=Veracruz, NL=Nuevo León, Pue=Puebla; Col=Colombia, Pan=Panama; FG=French Guyana.**
