# Supplementary material for: Appearance may be deceiving: Mexican sand flies (Diptera: Psychodidae: Phlebotominae) embrace a high diversity of cryptic species
Source: J Insect Sci. 2025 Jul 25;25(4):4. doi: 10.1093/jisesa/ieaf070 (PMC12290217; doi:10.1093/jisesa/ieaf070)
Supplement: ieaf070_suppl_Supplementary_Tables_S5 [file ieaf070_suppl_supplementary_tables_s5.doc]

**Appearance may be deceiving: Mexican sand flies (Diptera: Psychodidae: Phlebotominae) embrace a high diversity of cryptic species**

**Supplementary Table S5. *Fst* values for sand fly populations distributed between USA, Mexico, Panama Ecuador and Colombia.**

| P1 | P2 | *Fst* |
| --- | --- | --- |
| *Mi*. *cay*. *cayennesis* (ColI) | *Mi*. *cay*. *cayennesis* (ColII/Pan) | 0.78 |
| *Mi*. *cay*. *cayennesis* (ColII/Pan) | *Mi*. *cay*. *maciasi* | 0.96 |
| *Mi*. *durani* sp.1 | *Mi*. aff. *durani* sp.2 | 0.84 |
| *Mi*. *durani* sp.1 | *Mi*. *cay*. *cayennesis* (ColII/Pan) | 0.88 |
| *Mi*. *durani* sp.2 | *Mi*. *cay*. *cayennesis* (ColII/Pan) | 0.94 |
| *Pa*. *shannoni* (NL) | *Pa*. *shannoni* (QR) | 0.91 |
| *Pa*. *shannoni* (NL) | *Pa*. *shannoni* (Ver) | 0.99 |
| *Pa*. *shannoni* (QR) | *Pa*. *shannoni* (Ver) | 0.96 |
| *Pa*. *shannoni* (QRI) | *Pa*. *shannoni* (QRII) | 0.86 |
| *Pa*. *texana* (Mex) | *Pa*. *texana* (USA) | 0.58 |
| *Pa*. *carpenteri* (Ver) | *Pa*. *carpenteri* (QR) | 0.88 |
| *Pa*. *carpenteri* (Ver) | *Pa*. *carpenteri* (ColII/Pan) | 0.86 |
| *Pa*. *carpenteri* (QR) | *Pa*. *carpenteri* (ColII/Pan) | 0.95 |
| *Pa*. *carpenteri* (ColI) | *Pa*. *carpenteri* (ColII/Pan) | 0.96 |
| *Pa*. *carpenteri* (ColI) | *Pa*. *carpenteri* (Ver) | 0.92 |
| *Pa*. *carpenteri* (ColI) | *Pa*. *carpenteri* (QR) | 0.97 |
| *Lu*. *cruciata* (NL) | *Lu*. *cruciata* (Ver) | 0.69 |
| *Lu*. *cruciata* (NL) | *Lu*. *cruciata* (QR) | 0.37 |
| *Lu*. *cruciata* (QR) | *Lu*. *cruciata* (Ver) | 0.30 |
| *Lu*. *cruciata* (ChiI) | *Lu*. *cruciata* (Ver) | 0.55 |
| *Lu*. *cruciata* (ChiI) | *Lu*. *cruciata* (QR) | 0.12 |
| *Lu*. *cruciata* (ChiI) | *Lu*. *cruciata* (NL) | 0.48 |
| *Lu*. *cruciata* (ChiII/Col) | *Lu*. *cruciata* (NL) | 0.81 |
| *Lu*. *cruciata* (ChiII/Col) | *Lu*. *cruciata* (Ver) | 0.86 |
| *Lu*. *cruciata* (ChiII/Col) | *Lu*. *cruciata* (QR) | 0.75 |
| *Lu*. *cruciata* (ChiII/Col) | *Lu*. *cruciata* (ChiI) | 0.77 |
| *Pi*. *ovallesi* (Qr and Tab) | *Pi*. *ovallesi* (Pan) | 0.84 |
| *Pi*. *ovallesi* (Qr and Tab) | *Pi*. *ovallesi* (Chi) | 0.94 |
| *Pi*. *ovallesi* (Pan) | *Pi*. *ovallesi* (Chi) | 0.66 |
| *Ps*. *panamensis* (Mex) | *Ps*. *panamensis* (Ecu/Col I) | 0.61 |
| *Ps*. *panamensis* (Mex) | *Ps*. *panamensis* (Ecu/Col II) | 0.6 |
| *Ps*. *panamensis* (Mex) | *Ps*. *panamensis* (Pan) | 0.67 |
| *Ps*. *panamensis* (Pan) | *Ps*. *panamensis* (Ecu/Col I) | 0.35 |
| *Ps*. *panamensis* (Pan) | *Ps*. *panamensis* (Ecu/Col II) | 0.57 |
| *Ps*. *panamensis* (Ecu/Col I) | *Ps*. *panamensis* (Ecu/Col II) | 0.5 |
