# Supplementary figures and images for: Appearance may be deceiving: Mexican sand flies (Diptera: Psychodidae: Phlebotominae) embrace a high diversity of cryptic species
Source: J Insect Sci. 2025 Jul 25;25(4):4. doi: 10.1093/jisesa/ieaf070 (PMC12290217; doi:10.1093/jisesa/ieaf070)

rate

1.0

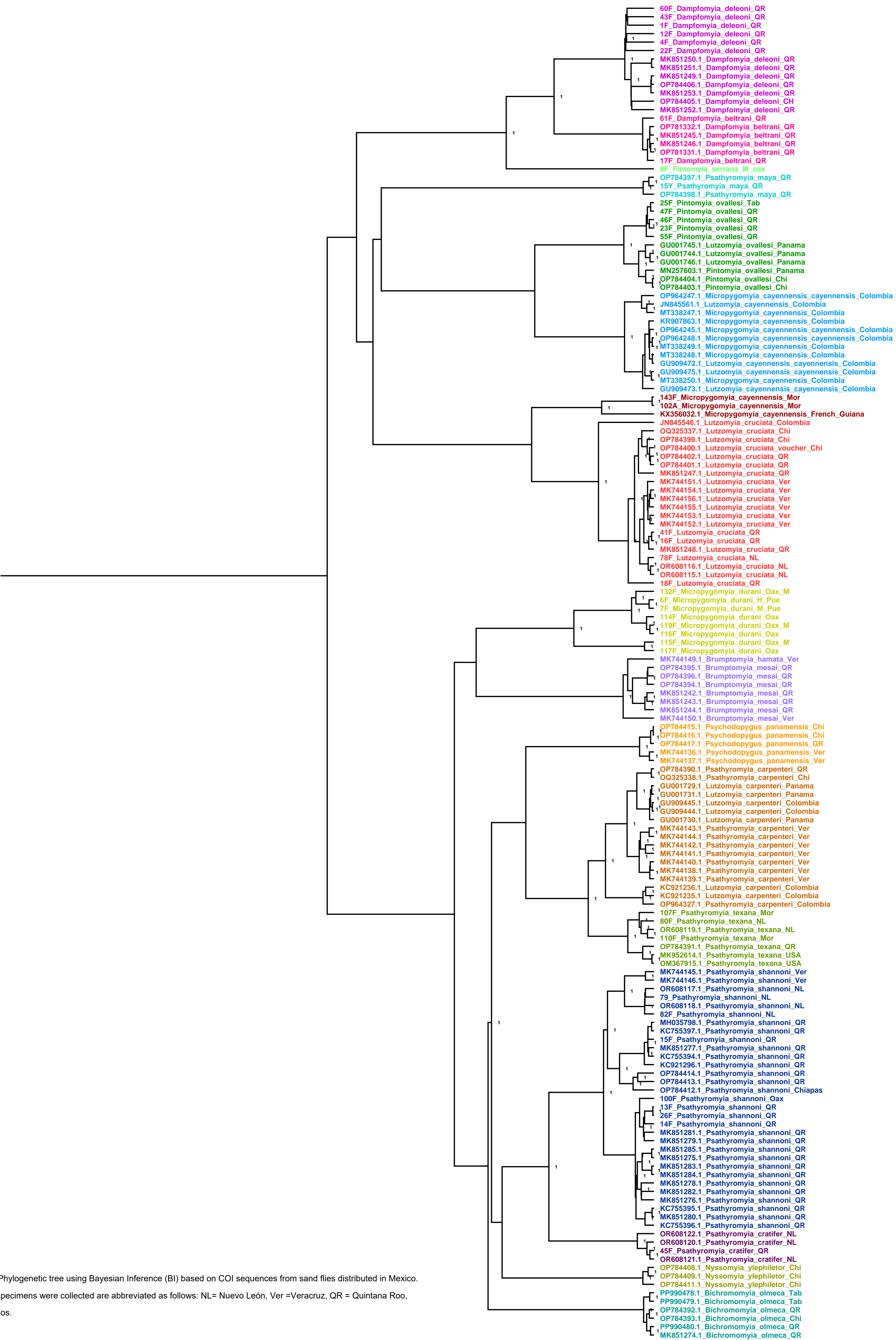

Supplement: ieaf070_suppl_Supplementary_Figures_S1 [file ieaf070_suppl_supplementary_figures_s1.pdf]

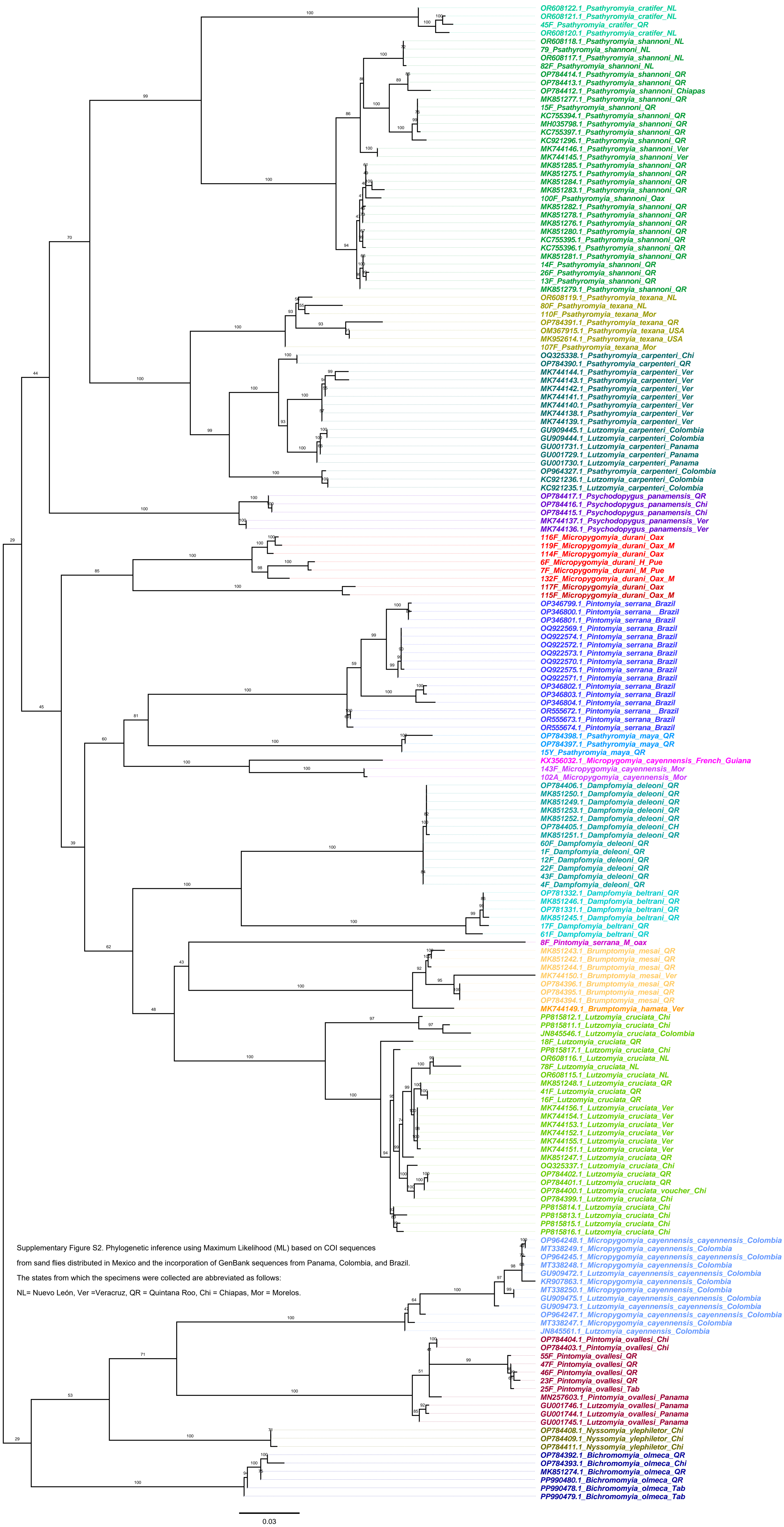

Supplement: ieaf070_suppl_Supplementary_Figures_S2 [file ieaf070_suppl_supplementary_figures_s2.pdf]
